# Functional and structural neuroplasticity of somatosensory system in hemiplegic cerebral palsy

**DOI:** 10.1093/braincomms/fcag185

**Published:** 2026-05-21

**Authors:** Sadra Shahdadian, Yanlong Song, Alireza Vaysi, Setu Shiroya, Haden Ray, Margherita A G Matarrese, Madhan Bosemani, Fernando Acosta, Brooke Kimbrell, Warren Marks, Christos Papadelis

**Affiliations:** Neuroscience Research, Jane and John Justin Institute for Mind Health, Cook Children's Health Care System, Fort Worth, TX 76104, USA; Department of Bioengineering, University of Texas at Arlington, Arlington, TX 76010, USA; Departments of Physical Medicine and Rehabilitation and Radiology, University of Texas Southwestern Medical Center, Dallas, TX 75390, USA; Neuroscience Research, Jane and John Justin Institute for Mind Health, Cook Children's Health Care System, Fort Worth, TX 76104, USA; Department of Bioengineering, University of Texas at Arlington, Arlington, TX 76010, USA; Burnett School of Medicine, Texas Christian University, Fort Worth, TX 76129, USA; Department of Kinesiology, Texas Christian University, Fort Worth, TX 76109, USA; Neuroscience Research, Jane and John Justin Institute for Mind Health, Cook Children's Health Care System, Fort Worth, TX 76104, USA; Department of Engineering, Università Campus Bio-Medico di Roma, Rome 00128, Italy; Neuroscience Research, Jane and John Justin Institute for Mind Health, Cook Children's Health Care System, Fort Worth, TX 76104, USA; Neuroscience Research, Jane and John Justin Institute for Mind Health, Cook Children's Health Care System, Fort Worth, TX 76104, USA; Neuroscience Research, Jane and John Justin Institute for Mind Health, Cook Children's Health Care System, Fort Worth, TX 76104, USA; Neuroscience Research, Jane and John Justin Institute for Mind Health, Cook Children's Health Care System, Fort Worth, TX 76104, USA; Department of Pediatrics, University of North Texas Health Science Center, Fort Worth, TX 76107, USA; Neuroscience Research, Jane and John Justin Institute for Mind Health, Cook Children's Health Care System, Fort Worth, TX 76104, USA; Department of Bioengineering, University of Texas at Arlington, Arlington, TX 76010, USA; Burnett School of Medicine, Texas Christian University, Fort Worth, TX 76129, USA

**Keywords:** hemiplegic cerebral palsy, neuroplasticity, electromagnetic source imaging, diffusion MRI

## Abstract

Children with hemiplegic cerebral palsy exhibit diverse sensory deficits linked to varied atypical brain reorganization patterns. The underlying mechanisms of these patterns remain poorly understood. Using multimodal neuroimaging, we associate functional and structural neuroplasticity in the somatosensory system of children with hemiplegic cerebral palsy with lesion type and behavioural outcome. We hypothesize that different lesion types produce distinct patterns of neuroplasticity, which correlate with varying levels of sensory and motor behavioural deficits. We examined 22 children with hemiplegic cerebral palsy (7 with cortical-subcortical, 12 with periventricular, and 3 with other lesions) and 24 age-matched neurotypical controls. We assessed the somatosensory outcomes through touch sensitivity and two-point discrimination measures, and motor outcomes through movement range, accuracy, dexterity, and fluency measures. We quantified the integrity of ascending sensory and somatosensory commissural fibres with diffusion-weighted MRI and measured cortical responses to haptic stimulation with magnetoencephalography and high-density EEG. Children with cortical-subcortical lesions demonstrated higher sensory thresholds and worse movement quality in the paretic hand compared to the periventricular lesion (*P* *<* 0.05) and control group (*P* *<* 0.01). Functional imaging revealed suppressed primary and secondary somatosensory cortical activations (*P* *<* 0.01) and prolonged primary somatosensory cortex latency (*P* *<* 0.05) in the more affected hemisphere in response to paretic hand stimulation. These children also exhibited more pronounced disruption of interhemispheric functional balance (*P* *<* 0.05). Tractography showed greater microstructural damage—increased mean (*P* *<* 0.01), axial (*P* *<* 0.01), and radial (*P* *<* 0.01) diffusivities—in both ascending sensory and commissural fibres in the more affected hemisphere of the cortical-subcortical lesion group. In contrast, the periventricular lesion group showed unilateral damage to ascending sensory fibres (*P* *<* 0.01) and bilateral damage to commissural fibres (*P* *<* 0.05). More severe diffusion abnormalities were associated with lower cortical amplitudes (*Rho* = −0.58 to –0.78, *P* *<* 0.05), delayed latencies (*Rho* = 0.69, *P* *<* 0.05), and decreased lateralization (*Rho* = −0.62 to −0.72, *P* *<* 0.05). Moderate to strong correlations were observed between cortical amplitudes and touch sensitivity (*Rho* = −0.56 to −0.60, *P* *<* 0.05) and discriminability (*Rho* = –0.77, *P* *<* 0.01). Our findings show that children with hemiplegic cerebral palsy exhibit distinct structural and functional neuroplasticity patterns based on lesion type. Cortical-subcortical lesions lead to more extensive somatosensory damage, while periventricular lesions are linked to greater neuroplastic potential. These insights deepen our understanding of neuroplasticity mechanisms and highlight targets for optimizing therapeutic interventions.

## Teaser

With multimodal imaging, Shahdadian *et al*. identified distinct neuroplasticity patterns in the somatosensory system of children with hemiplegic cerebral palsy depending on lesion type. Cortical-subcortical lesions lead to extensive somatosensory damage, while periventricular lesions have greater neuroplastic potential.

## Graphical Abstract

**Graphical Abstract:**
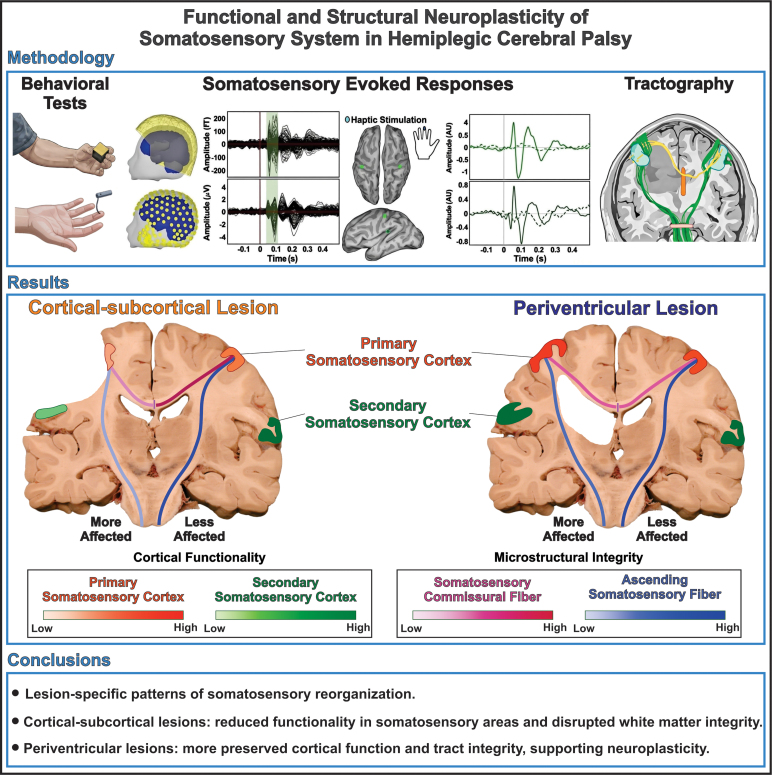

## Introduction

Cerebral palsy encompasses a variety of neurodevelopmental disorders, mainly manifesting as movement and postural abnormalities, often associated with sensory deficits.^1-4^ The most predominant cerebral palsy form is hemiplegic cerebral palsy (HCP),^5,6^ which is characterized by heterogeneous levels of motor and somatosensory deficits on one side of the body, often affecting the hand functions and bimanual coordination.^7^ The severity of these deficits is influenced by several factors, including the underlying aetiology, timing of the insult, and the extent to which the affected physiological system(s) respond to them. HCP is often caused by a non-progressive brain insult that occurs unilaterally during the perinatal period.^8^ The brain's response to this insult is adaptive neuroplasticity through the formation of new neural connections.^9-14^ The extent and effectiveness of this neuroplasticity vary widely among individuals, since it is influenced by the timing and size of the lesion as well as the involved circuit(s).^15,16^ Despite the progress made to date, our understanding of adaptive neuroplasticity, particularly in the somatosensory system, remains limited, and the neural correlates of various neuroplasticity patterns remain unknown.

We currently lack a complete understanding of how aetiology is associated with functional and structural neuroplasticity as well as behavioural outcomes. This lack of knowledge has led to generic, low-impact therapy approaches that fail to augment the individual’s neuroplasticity potential. Thus, there is an unmet need for individualized neuroplasticity mapping, especially for children with severe somatosensory deficits who commonly exhibit higher levels of hand impairments.^11^ Aiming to link the sensorimotor deficits with aetiology, previous MRI studies categorized most children with HCP into either having periventricular (PV) or cortical-subcortical (CSC) lesions.^17,18^ PV lesions mainly occur in the early third trimester of gestation and are characterized by white matter volume loss and compensatory ventricular dilation.^19,20^ Contrarily, most CSC lesions typically occur in the late third trimester and postnatal period;^21^ these lesions cause volume loss of the cortical and subcortical grey matter, as well as the adjacent white matter.^22^ Children with CSC lesions experience more pronounced upper extremity impairments, particularly in the somatosensory function, than those with PV lesions.^11,18,23,24^ These differences in behavioural outcomes may be determined partially by the timing of the insult. For instance, the occurrence of PV insults before the development of ascending somatosensory fibres (ASF) usually leads to these fibres bypassing the lesion and reaching the spared cortex. In contrast, CSC lesions commonly damage the already developed ASF and somatosensory cortices that disrupt the transfer and processing of afferent information, respectively.^17,25^ To underscore the effect of perinatal brain insult on white matter fibres, previous diffusion-weighted imaging (DWI) studies have examined the microstructural integrity of ASF and found indications of damage within the more affected (MA) hemisphere.^11,13,14,18^ Other DWI studies have shown that microstructural damages are also extended to the association and commissural fibres.^14,26^ Despite the structural information provided by DWI, this technique does not offer insights on possible alterations in the functionality of the underlying somatosensory circuitry.

Functional neuroimaging methods, such as event-related potentials and fields (ERP and ERF, respectively), provide critical insights into the sensorimotor circuit functionality. By employing functional neuroimaging techniques, our group and others have shown alterations in the somatosensory evoked potentials/fields (SEP/SEF) of children with HCP, such as changes in amplitude, somatotopy, and phase synchronization between the primary (S1) and secondary (S2) somatosensory cortices.^12,13,27,28^ These findings reveal maladaptive changes in the function of different nodes in the somatosensory circuit (e.g. S1 and S2),^12,13,28^ and distorted information transmission between them.^28^ Stratifying different lesion types and their distinct effect on this circuit’s functionality may explain variability in behavioural outcomes and aid in tailoring future rehabilitation approaches.^25,29^

By employing multimodal neuroimaging, we aim to identify atypical patterns of functional and structural somatosensory reorganization in the brain of children with HCP and their association with the lesion type and behavioural outcome. We hypothesize that different lesion types produce distinct neuroplasticity patterns, which correlate with varying levels of somatosensory and motor behavioural deficits. To assess the functional alterations in the somatosensory circuit and interhemispheric balance, we recorded simultaneously ERP and ERF in response to haptic stimulation and localized S1 and S2 through electromagnetic source imaging^30^ in a cohort of children with HCP and a group of neurotypically developing controls. In contrast to prior studies, this study employs combined MEG and HD-EEG with superior spatial resolution^30-32^ to identify lesion-adjacent cortical sources and to quantify source-level response dynamics, including amplitude, latency, and interhemispheric imbalance. Importantly, these functionally localized cortical regions were subsequently used as anatomically informed seeds for DWI tractography, enabling the investigation of microstructural integrity within the ASF and the previously underexplored somatosensory commissural fibres (SCF). Using diffusion-derived microstructural metrics, we examined how alterations in these pathways relate to functional reorganization and behavioural performance. We classified most HCP children in our cohort into two groups (i.e. CSC and PV groups) based on lesion type. We then examined associations between functional neuroplasticity, structural alterations in the ASF and SCF, and sensorimotor behavioural outcomes across groups.

## Materials and methods

### Participants

We recruited 22 children with HCP (15 males, 7 females; 10.8 ± 3.2 years) and 24 typically developing (TD) children (13 males, 11 females; 11.0 ± 3.4 years). Children with HCP were recruited from the movement disorders clinic at Cook Children’s Medical Center (CCMC), Fort Worth, TX. TD children were recruited from the local community. The inclusion criteria for the HCP participants were the following: (1) evaluation by a paediatric neurologist, neonatal developmental specialist, or neonatologist with an HCP diagnosis; (2) classified as high-functioning (GMFCS I or II); and (3) age-appropriate understanding of study procedures. The exclusion criteria were as follows: (1) psychoactive or myorelaxant medication during study procedures; (2) genetic syndrome diagnosis; (3) history of trauma or brain surgery; (4) inability to sit still; and (5) metal implants or baclofen pumps in the body. TD children had no neurological disorder or brain injury. The Institutional Review Board at Cook Children’s Health Care System approved this study (2019-068; PI: C. Papadelis).

### Behavioural assessment

#### Touch sensitivity and discrimination

**Figure 1:**
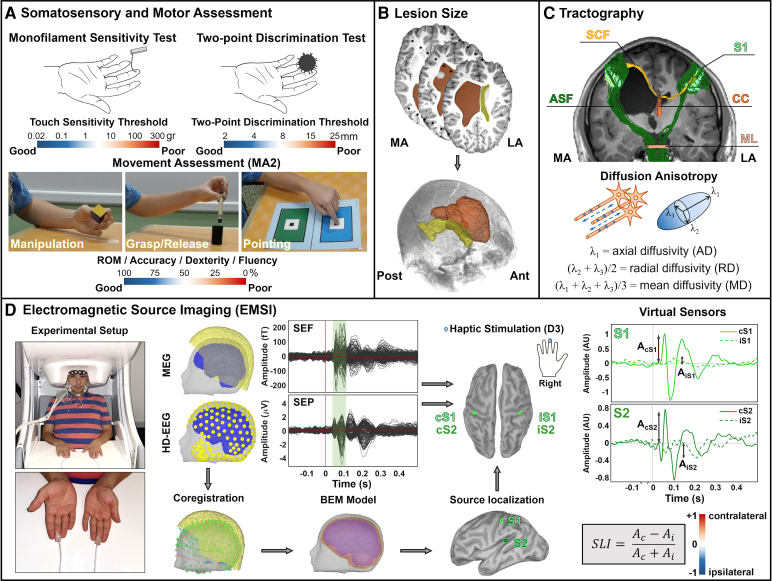
**Overview of data collection and analysis.** (**A**) Somatosensory and motor assessments, including monofilament sensitivity, two-point discrimination and movement quality evaluation. (**B**) Reconstruction of bilateral ventricular volumes from T1-weighted MRI of a participant with a periventricular (PV) lesion (#9). Lesion/ventricular dilation size quantified by subtracting ventricular volume in the less affected (LA) from the more affected (MA) hemisphere. (**C**) Tractography derived from diffusion-weighted imaging (DWI). Anatomically defined (corpus callosum [CC], medial lemniscus [ML]) and functionally guided (primary somatosensory cortex [S1]) regions of interest were used to isolate ascending sensory (ASF) and somatosensory commissural fibres (SCF). Diffusion metrics—axial diffusivity (AD), radial diffusivity (RD) and mean diffusivity (MD)—were extracted from each tract. (**D**) Electromagnetic source imaging experimental setup and analysis pipeline. Photograph of the magnetoencephalography (MEG) and high-density EEG (HD-EEG) experimental setup is shown depicting an adult volunteer placing his head inside the MEG helmet with pneumatic haptic stimulators attached to his fingertips; this photograph is provided for demonstration purposes and does not represent a study participant. MEG (306 sensors) and HD-EEG (256 channels) data extracted somatosensory evoked fields (SEF) and potentials (SEP) at the sensor level. MEG and EEG sensor locations were coregistered onto the subject’s 3D head and cortical surfaces. A realistic boundary element method (BEM) head model constructed from the subject’s T1-weighted MRI. The inverse model localized activity in bilateral primary (S1) and secondary (S2) somatosensory cortices from haptic stimulation of the middle finger (D3). Virtual sensors in bilateral S1 and S2 provided cortical activity time series. Amplitude and latency of peak activity computed in both S1 and S2. Somatosensory lateralization index (SLI) calculated for S1 and S2 to assess interhemispheric balance between contralateral and ipsilateral responses. A, amplitude; Ant, anterior; c, contralateral; i, ipsilateral; MA2, Melbourne Assessment 2; Post, posterior.

We tested touch sensitivity on the thumb (D1), middle finger (D3), and pinky (D5) of both hands using Semmes–Weinstein monofilaments^33^ (Fig. 1A). The monofilaments were applied in a random order while the participant had his/her eyes closed. For each stimulus, participants reported perception. Each monofilament was applied three times per finger. A touch sensitivity threshold was determined for each finger as the lowest weight perceived twice.

We also tested static two-point discrimination using the Touch Test® Two-Point discriminator^34^ (Exacta™ Evaluation Kit; North Coast Medical Inc., CA). The discrimination thresholds were determined on D1, D3 and D5 of both hands three times by applying either one- or two-point stimulus to fingertips randomly, while the participant had his/her eyes closed. The participant was asked to state whether he/she perceived one- or two-point stimulus. A discrimination threshold was determined for each finger as the lowest distance perceived twice.

### Movement quality

We evaluated unilateral upper-extremity function, including range of motion, accuracy, dexterity, and fluency, using the Melbourne Assessment 2^35^ (Fig. 1A). Children were given various items to manipulate and were scored differently related to each movement element. A total of 30 item scores was used. Scoring was conducted by two double-blinded researchers (S.S. and E.A.) and was completed across the 30 score items using a 3, 4, or 5 scale and individually defined scoring criteria. Item scores relating to each element measured were summed within the corresponding subscale.

### MRI/DWI acquisition

The imaging protocol included structural and DWI sequences collected at Radiology of CCMC. We obtained T1-weighted structural MRI scans using a Siemens Skyra 3T MR scanner and a 10-channel head coil with 3D magnetization-prepared rapid-acquisition gradient-echo sequence with parameters: echo time = 3.22 ms, inversion time = 450 ms, repetition time = 8.21 ms, flip angle = 15 degrees, field of view = 24 cm, matrix size = 256 × 256, 184 slices, and resolution = 1.0 × 1.0 × 1.0 mm^3^. DWI employed a multi-shell diffusion scheme (b-values: 2000 s/mm^2^) with diffusion sampling directions of 30 and 60, respectively. The in-plane resolution was 2.04918 mm, and the slice thickness was 2 mm.

### Lesion type, affected hemispheres, and structures

A paediatric neuroradiologist (M.B.) reviewed the MRI scans. Together with a paediatric neurologist (W.M.), they identified the underlying HCP lesion type, as well as the affected hemisphere and cortical/subcortical structures. These structures include cortical grey matter, periventricular white matter, basal ganglia, thalamus, and corpus callosum. For all HCP participants, the hemisphere with the most pronounced structural abnormalities was labelled as MA. Paretic hand (PH) was contralateral to the MA hemisphere in all HCP participants. Clinical characteristics of HCP participants are in Table 1. In TD participants, we marked the contralateral hemisphere to the dominant hand (D-hand) as the dominant hemisphere (D-hemi).

**Table 1:** Demographics and clinical characteristics of hemiplegic cerebral palsy participants

#	Age [y]	Gender	Affected body side	MRI findings side)	Brain structures affected	Lesion type	Gestational age [w]	GMFCS	MACS	Functional neuroimaging
1	11	M	R	MCA stroke (L)	Cortex, WM	CSC	40	2	2	MEG, HD-EEG
2	16	M	R	MCA stroke (L)	Cortex, WM, BG, thalamus	CSC	37	1	5	MEG
3	8	F	L	IVH (R: IV, L: II)	Cortex, WM, CC, thalamus	PV	26	1	2	MEG, HD-EEG
4	12	M	R	HIE (L)	WM, CC, BG, thalamus	PV	37	2	1	MEG, HD-EEG
5	11	M	R	MCA stroke (L)	Cortex, WM, thalamus	CSC	41	2	1	MEG, HD-EEG
6	10	M	L	IVH (R: IV)	WM, CC, BG, thalamus	PV	24	2	2	MEG, HD-EEG
7	5	M	L	MCA stroke (R)	Cortex, WM, BG, thalamus	CSC	38.5	2	5	MEG, HD-EEG
8	6	F	R	PVL (L > R)	WM, BG, thalamus	PV	33	1	1	MEG, HD-EEG
9	14	M	R	IVH (L: IV)	WM, BG, CC	PV	26	2	3	MEG, HD-EEG
10	10	F	L	Polymicrogyria (R)	Cortex	other	39	1	3	MEG
11	12	M	L	Polymicrogyria pachygyria (R)	Cortex	other	36	2	2	MEG, HD-EEG
12	9	M	R	MCA stroke (L)	Cortex, WM, thalamus	CSC	41	1	3	MEG, HD-EEG
13	13	F	R	MCA stroke (L)	Cortex, WM, CC, BG, thalamus	CSC	40	2	3	MEG
14	12	M	R	MCA stroke (L)	Cortex, WM, CC, BG, thalamus	CSC	38	2	5	MEG, HD-EEG
15	10	M	R	IVH (L: II)	WM	PV	37	1	1	MEG, HD-EEG
16	14	F	R	Schizencephaly, polymicrogyria (L)	Cortex, WM	other	-	2	3	MEG, HD-EEG
17	15	M	L	IVH (R: IV)	WM, CC, BG, thalamus	PV	36	1	3	MEG, HD-EEG
18	8	M	R	IVH (L: II)	WM, CC	PV	39	2	2	MEG, HD-EEG
19	15	M	L	HIE—PVL (R > L)	WM	PV	29	1	2	MEG
20	14	F	L	PVL (R > L)	WM	PV	26	2	1	MEG, HD-EEG
21	5	F	R	Centrum semiovale gliosis (L)	WM	PV	36	2	1	MEG
22	7	M	L	Periventricular WM gliosis(R)	WM	PV	37	1	1	MEG, HD-EEG

BG, basal ganglia; CC, corpus callosum; CSC, cortical-subcortical; EEG, electroencephalography.; F, female; GMFCS, gross motor function classification system; HIE, hypoxic-ischemic encephalopathy; IVH, intraventricular haemorrhage; L, left; M, male; MACS, manual ability classification system; MCA, middle cerebral artery; MEG, magnetoencephalography; PV, periventricular; PVL, periventricular leukomalacia; R, right; WM, white matter.

### Calculation of lesion/ventricular dilation size

We determined the lesion/ventricular dilation size from the axial T1-weighted MRI and verified it in coronal/sagittal views. The lesion borders were manually marked (A.V.) with the MATLAB® Volume Segmenter toolbox. The corresponding lesion/dilated ventricle volume was then computed (Fig. 1B). Lesion volume included regions of grey matter loss and ex-vacuo ventricular dilation secondary to white matter volume loss. To account for baseline ventricle size, we subtracted the average normal ventricular volume (obtained from the TD cohort) from the total volume of the lesion and dilated ventricle in the MA hemisphere.

### Acquisition of ERP/ERF

We performed simultaneous magnetoencephalography (MEG) and high-density electroencephalography (HD-EEG) recordings inside a single-layer magnetically shielded room located at suite Radiology of CCMC (Fig. 1D). The combination of MEG and EEG recordings enhances the accuracy of source localization by leveraging their complementary sensitivities. Previous studies showed that MEG is primarily sensitive to tangential cortical currents, and EEG is more robust in detecting radial components.^36^ This multimodal approach enables a more comprehensive characterization of somatosensory evoked responses, particularly in the presence of lesions or when investigating deeper cortical sources.^30-32^

MEG recordings were performed with a whole head 306 sensor system (VectorView, Elekta Neuromag, Helsinki, Finland). HD-EEG recordings were performed with the Geodesic EEG System 400 (Magstim EGI Inc., USA) that uses nets with either 128 or 256 channels (depending on participant’s head size). More particularly, in the TD group, we used the 128-channel net for one and the 256-channel net for 19 participants. In the CP group, we used the 128-channel net for four and the 256-channel net for 13 participants. Four TD and 5 CP participants performed the test with MEG data collection only. Both 128- and 256-channel datasets were processed with the same analysis pipeline without using a 128-channel subset of channels in the 256-channel sets.

Before data acquisition, we placed five head position indicator coils on child's head, and used a digitizer (Fastrak Polhemus, USA) to map head cardinal landmarks and ∼500 scalp points into digital 3D coordinates. This enabled alignment of MEG sensors with participant’s MRI for source localization. We also recorded electrocardiography (ECG) and electrooculography (EOG) signals and later used them for the removal of artefacts from heartbeats and blinks on MEG signal, respectively. Before magnetically shielded room entry, we positioned the EEG net on the participant’s head and checked the electrode impedances to ensure adequate conductivity (impedance < 50 KΩ). To determine the electrode positions with respect to the participant's head, we used the GeoScan Sensor Digitization system (Magstim EGI Inc., USA). When simultaneous MEG and HD-EEG were not feasible, we collected only MEG data (Table 1).

As shown in Fig. 1D, for SEF and SEP recordings, we delivered compressed air bursts to middle fingertips (D3) through a pneumatic stimulation system (AIRSTIM, San Diego Instruments, USA). We applied stimulations asynchronously with a 1.5 ± 0.5-s interstimulus interval following a pseudorandom sequence (400 stimuli per finger). The duration of the air bursts was 10 ms. The air bursts were applied to the finger pads, while the hands were positioned palm down on the tray. Participants kept their arms motionless on a tray and watched visually engaging but hand-neutral cartoons to minimize movement artefacts, which ensures preserving SEF and SEP characteristics.^37^ The cartoons did not include hand- or action-related content, minimizing the risk of mirror-neuron system activation and associated confounds.

### Electromagnetic data analysis

We processed the raw MEG signals with MaxFilter (version 2.2.10, MEGIN, Finland) employing the temporal extension of signal-space separation (tSSS) to reduce noise and correct for head motions. The following parameters were used in the MaxFilter software: buffer length 10 s, subspace correlation limit 0.98, inside expansion order 8, and outside expansion order 3. Furthermore, an in-house algorithm^30^ spatially aligned and temporally synchronized the MEG/HD-EEG data. We manually removed MEG and HD-EEG bad channels, including the HD-EEG channels located on the face and neck. We then performed baseline correction (DC offset removal), band-pass (1–100 Hz), and notch (60 Hz) filters on MEG and HD-EEG data using *Brainstorm* (version October 2024).^38^ Band-pass filtering was performed using an even-order, linear-phase finite impulse response filter with a Kaiser window design. The filter order was estimated using MATLAB’s *kaiserord* function, and the filter was implemented with *fir1*. The stopband attenuation was set to 60 dB. Line noise was attenuated using a second-order infinite impulse response (IIR) notch filter with zero-phase lag (implemented with *filtfilt*), a bandwidth of 3 Hz, and a stopband attenuation of 60 dB. We then removed artefacts through signal-space projection (SSP) and independent component analysis (ICA) methods^39,40^ for the MEG and HD-EEG data, respectively. MEG artefacts caused by heartbeats and blinks were automatically detected from ECG and EOG recordings and then removed with the SSP application. HD-EEG artefacts caused by heartbeats, eye movements, blinks, and muscle contraction were identified and removed with the application of ICA. For the data with excessive muscle contraction artefact (e.g. children with spasticity), we identified HD-EEG and MEG channels with prominent muscle activity. We then removed the independent components with the highest correlations with those channels. Lastly, we inspected and removed bad segments of MEG and HD-EEG data and their corresponding trials. While preprocessing steps such as filtering and artefact correction parameters were standardized across participants, certain procedures including ICA component rejection and identification of bad segments involved expert-guided decisions, reflecting a combination of objective and subjective elements inherent to electrophysiological data analysis pipelines.

#### Source localization

To obtain SEF and SEP evoked by finger stimulation, we segmented continuous sensor data into event-locked trials of 700 ms (200 pre- and 500 ms post-stimulus). We calculated grand averages by averaging trials for each participant and removed the channels with excessive activity in the pre-stimulus segment of the SEF or SEP.

Dynamic statistical parametric mapping (dSPM)^41^ was employed to identify neural sources.^42^ We generated canonical surfaces (i.e. cortex, inner skull, outer skull and scalp) from individual T1-weighted MRI data. We then co-registered MEG and HD-EEG signals by projecting digitized cranial locations onto scalp’s surface and estimated the forward models using the boundary element modelling (BEM) with *OpenMEEG*.^43^ We derived noise covariance matrices from pre-stimulus activity in the −200 to −10 ms window. Using *Brainstorm’s* default parameters, we computed the dSPM source activity for ∼15 000 cortical vertices, incorporating gradiometers, magnetometers, and HD-EEG channels. Default dSPM settings were retained: regularization of noise covariance by adding 10% of its largest eigenvalue with signal-to-noise (SNR) ratio of 3, constrained dipole orientations normal to the cortical surface, and z-score normalization of source maps relative to the noise covariance. We identified peak cortical activities corresponding to S1 and S2, located in Brodmann’s areas 3/1/2 and 43, respectively. We extended the S1 cortical vertices to surrounding vertices for defining an area of ∼3 cm^2^ and exported the coordinates as region of interest (ROI) for tractography (Fig. 1D). Due to severe cortical damage in two participants from the CSC group, the locations of the first and second SER peaks in response to haptic stimulation of the PH did not align with the anatomical atlas. Therefore, we selected the vertices with peak amplitude on the MA side as S1 and S2.

#### Somatosensory lateralization index

To assess functional interhemispheric balance between lateral S1 and S2, we computed the cortical somatosensory lateralization index (*SLI*) for the TD, CSC, and PV groups in response to each hand. We placed virtual sensors on bilateral neuroimaging-guided S1 and S2 cortical vertices to extract somatosensory evoked responses (SER) during lateral finger stimulation (Fig. 1D). We baseline-normalized the maximum activation value in each area and its contralateral counterpart and calculated SLI as:^44^

(1) $$SLI=\;\frac{A_{c}-A_{i}}{A_{c}+A_{i}}$$

where *A_c_* and *A_i_* represent amplitude of SER in contralateral and ipsilateral hemispheres, respectively. *SLI* ranges from −1 (ipsilateral activity) to +1 (contralateral activity).

### ROI-based tractography

We performed DWI preprocessing and tractography using *DSI-Studio* (version October 10, 2024). Our preprocessing steps included visual quality inspection, motion correction, and the creation of a white matter mask. We performed fibre tracking using Generalized Q-sampling imaging,^45^ which allows for the identification of complex white matter configurations, including crossing fibres. We set the threshold for tract angle to 60 degrees, step sizes from 0.5 to 1.5 voxels, and track lengths from 30 to 200 mm. We isolated a total of 3000 tracts and applied topology-informed pruning^46^ over 16 iterations to eliminate false connections.

We loaded primary somatosensory areas derived from functional neuroimaging into *DSI-Studio* and identified the ASF by setting S1 and the medial lemniscus as ROIs (Fig. 1C). We also defined SCF, which pass through the corpus callosum (CC), by setting unilateral S1 and the CC as ROIs. We then trimmed the identified SCFs to retain only the segments located in the hemisphere ipsilateral to the selected S1. In addition, generalized Q-sampling imaging allowed us to estimate diffusivity parameters [i.e. mean diffusivity (MD), axial diffusivity (AD) and radial diffusivity (RD)] of the reconstructed fibres to assess white matter microstructural integrity.

### Statistical analysis

An a priori power analysis using G*Power software determined that a total of 42 participants is needed to detect lesion-specific effects with medium effect size (Cohen’s f = 0.5), alpha = 0.05, and power = 0.8. To identify lesion-specific patterns of behavioural, neurophysiological, and microstructural alterations in the somatosensory network, we performed statistical analyses on data from TD participants and children with PV and CSC lesions. We used Wilcoxon signed rank tests to compare behavioural (i.e. sensitivity, discriminability, and Melbourne Assessment 2 parameters), functional (i.e. S1 and S2 amplitude, latency, and SLI), and microstructural (i.e. MD, AD, and RD) measures within hands/hemispheres of each group (i.e. TD, CSC, and PV groups). We also employed Kruskal–Wallis tests to compare these measures among two sets of data: (i) non-dominant hand (ND-hand)/hemisphere (ND-hemi) of TD versus paretic hand (PH)/MA of CSC and PV group and (ii) D-hand/D-hemi of TD versus non-paretic hand (NPH)/LA of CSC and PV groups. The significance level was set at 0.05. For post hoc testing, we performed pairwise Mann–Whitney *U* tests with false discovery rate (FDR) correction for multiple comparisons. Effect sizes were further calculated as r = |Z|/sqrt(N) derived from the normal approximation of Wilcoxon signed rank and Mann–Whitney *U* tests (for paired tests *N* = *n*, and for independent tests *N* = n_1_ + n_2_).

We used Spearman correlations to identify significant associations among behavioural outcomes, functional measures, and structural (i.e. lesion size, MD, AD and RD) measures. In these analyses, we aimed to examine associations between the (i) functional and behavioural measures; (ii) structural and behavioural measures; and (iii) structural and functional measures. To account for uneven sample sizes, we performed two sets of correlation analyses: one that included all conditions (i.e. both hands/hemispheres of the TD, CSC and PV groups), and another that included only the PH or the MA hemisphere of the CSC and PV groups. The significance level was set at 0.05. To correct for multiple comparisons, we performed FDR correction. All statistical analyses were conducted using R® version 4.5.0. Although data were collected from participants with other lesion types, these cases were not included in the statistical analyses due to heterogeneity and limited sample size. The corresponding results are reported in Supplementary Table 1 of the Supplementary Materials.

## Results

### Behavioural assessment

#### Touch sensitivity

**Figure 2:**
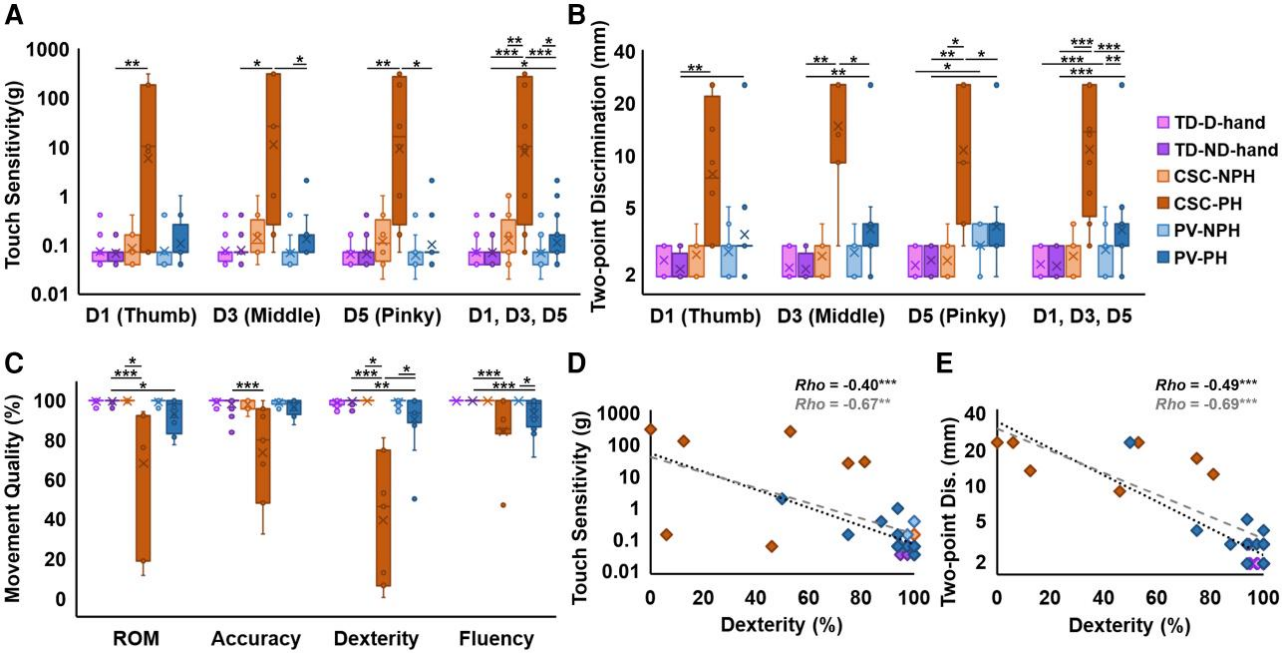
**Behavioural assessment results. (A)** Box-and-whisker plots of touch sensitivity thresholds (g, log scale) at thumb (D1), middle finger (D3), pinky (D5) and the pooled D1–D5 in the dominant (TD-D-hand) and non-dominant (TD-ND-hand) hands of typically developing (TD; *N* = 24) controls and the non-paretic (NPH) and paretic (PH) hands of children with cortical-subcortical (CSC; *N* = 7) or periventricular (PV; *N* = 12) lesions. (**B**) Static two-point discrimination thresholds (mm) in the same groups and digits. (**C**) Melbourne Assessment 2 sub-scores (%) for range of motion (ROM), accuracy, dexterity and fluency in the same groups. (**D, E**) Spearman correlations between dexterity sub-score and touch sensitivity (**D**) or two-point discrimination (**E**) in the PH, shown across all participants (black) and across PH of HCP participants only (grey). Boxes indicate median and interquartile range, whiskers range, × denotes mean, and o represents individual data points for each participant. *P < 0.05, **P < 0.01, ***P < 0.001 derived from Wilcoxon signed rank tests for within subject, and Kruskal–Wallis and Mann–Whitney *U* tests for between subject comparisons (FDR corrected for multiple comparisons).

Finger-level analysis revealed elevated touch sensitivity thresholds in the PH of the CSC group compared to ND-hand of TD participants (D1: *P* *<* 0.05, r = 0.30; D3: *P* *<* 0.05, r = 0.34; D5: *P* *<* 0.01, r = 0.38) and the PH of the PV group (*P* *<* 0.05, r > 2.01) (Fig. 2A). With pooled data from all three fingers, thresholds remained significantly higher in the PH of the CSC group compared to its NPH (*P* *<* 0.001, r = 0.88), PH of the PV group (*P* *<* 0.001, r = 2.01), and ND-hand of TD group (*P* *<* 0.001, r = 0.36). The PH of the PV group also showed higher thresholds than its non-paretic counterpart and ND-hand of TD group (*P* *<* 0.05, r = 0.19).

#### Two-point discrimination

Two-point discrimination thresholds were elevated in the PH of the CSC group compared to ND-hand of TD group (D1, D3, D5: *P* *<* 0.01, r > 0.39), as well as its NPH (D3, D5: *P* *<* 0.05, r = 0.89; Fig. 2B). Within the PV group, significant differences with small effects were observed between both hands and TD group (PH: D1: *P* *<* 0.01, r = 0.07, D3: *P* *<* 0.01, r = 0.13; D5: *P* *<* 0.05, r = 0.06; NPH: D5: *P* *<* 0.05, r = 0.04). Pooled three fingers, data demonstrated elevated thresholds in the PHs of CSC group (*P* *<* 0.001, r = 0.43) and both hands of PV group (*P* *<* 0.001, r = 0.1), relative to TD children. However, the PH of the PV group still showed lower thresholds than the CSC group (*P* *<* 0.001, r = 1.7).

#### Movement assessment

The PH of the CSC group exhibited reduced performance in all four domains of range of motion, accuracy, dexterity, and fluency, compared with its NPH and ND-hand of TD children (*P* *<* 0.001, r > 0.75; Fig. 2C). The PH of the PV group also showed decreased range of motion (*P* *<* 0.05, r = 0.78), dexterity (*P* *<* 0.01, r = 0.88), and fluency (*P* *<* 0.001, r = 0.84), relative to ND-hand of TD group. Strong negative correlations were observed between dexterity and hand-averaged touch sensitivity (*Rho* = −0.67, *P* *<* 0.01), as well as two-point discrimination thresholds (*Rho* = −0.69, *P* *<* 0.001) in the PHs of children with CSC and PV lesions (Fig. 2D and E).

### Anatomical alterations

All children with HCP had predominantly unilateral structural defects. Among the 22 children with HCP, 7 had CSC lesions (6 male, 11.0 ± 3.2 years), 12 had PV lesions (8 male, 10.3 ± 3.4 years), and 3 had other lesions (1 male, 12 ± 1.4 years). MRI review indicated white matter damage in all children with PV and CSC lesions. Cortical grey matter injury was seen in all CSC cases (7 of 7), but only in one PV case (1 of 12). CC thinning, primarily in the posterior body, was more frequently observed in the PV group (6 of 12) than in the CSC group (2 of 7). Basal ganglia and thalamic injuries were more common in CSC participants (basal ganglia: 4 of 7; thalamus: 6 of 7) compared with the PV group (basal ganglia: 5 of 12; thalamus: 5 of 12). MRI of TD children showed no structural abnormalities.

**Figure 3:**
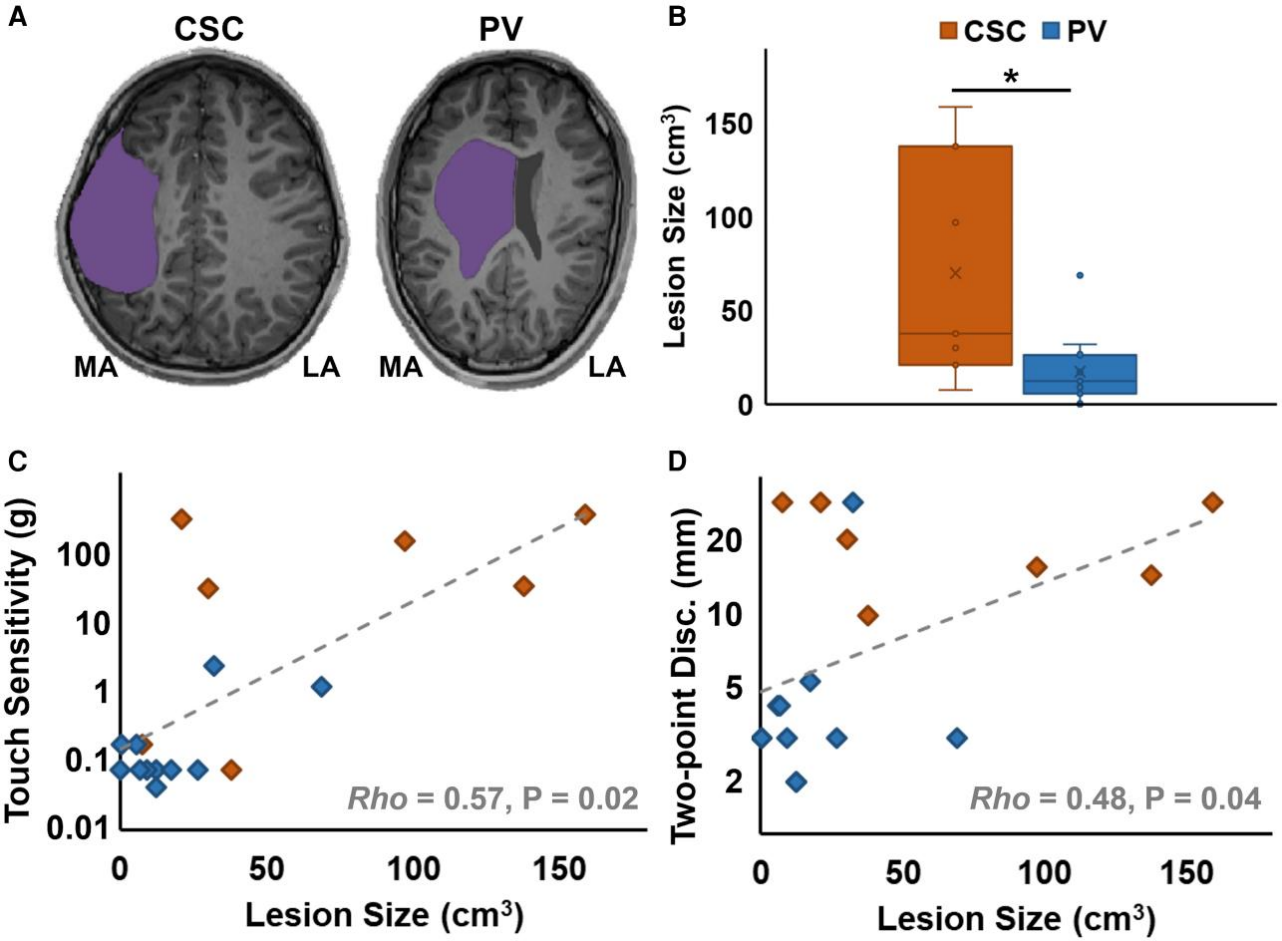
**Lesion/ventricular dilation volumes and their relation to sensory thresholds.** (**A**) Representative axial T1-weighted MRIs from cortical-subcortical (CSC; *N* = 7) and periventricular (PV; *N* = 11) groups with manual segmentations of enlarged ventricles/lesion cavities in the more affected (MA; purple) and less affected (LA; pink) hemispheres. (**B**) Box-and-whisker plot of lesion/ventricular dilation volumes (cm^3^) in CSC and PV groups. (**C, D**) Spearman correlations between lesion/ventricular dilation size in the MA hemisphere and touch sensitivity threshold (g; **C**) or two-point discrimination (mm; **D**) in the paretic hand (PH). Boxes in (**B**) indicate median and interquartile range, whiskers range, × denotes mean, and o represents individual data points for each participant. *P < 0.05, **P < 0.01 derived from Mann–Whitney *U* test and Spearman Correlation. ◊ in (**C**, **D**) represents individual data points for each participant.

Figure 3A shows examples of areas identified on MRIs of CSC and PV lesions. T1-weighted MRI analysis revealed larger lesion volumes in the CSC group compared with the PV group (*P* *<* 0.05, r = 1.95; Fig. 3B). Lesion/ventricular dilation size showed a positive correlation with PH touch sensitivity (*Rho* = 0.57, *P* *<* 0.05; Fig. 3C) and two-point discrimination thresholds (*Rho* = 0.48, *P* *<* 0.05), indicating that larger lesions are associated with more severe sensory impairments (Fig. 3D).

### SER features

**Figure 4:**
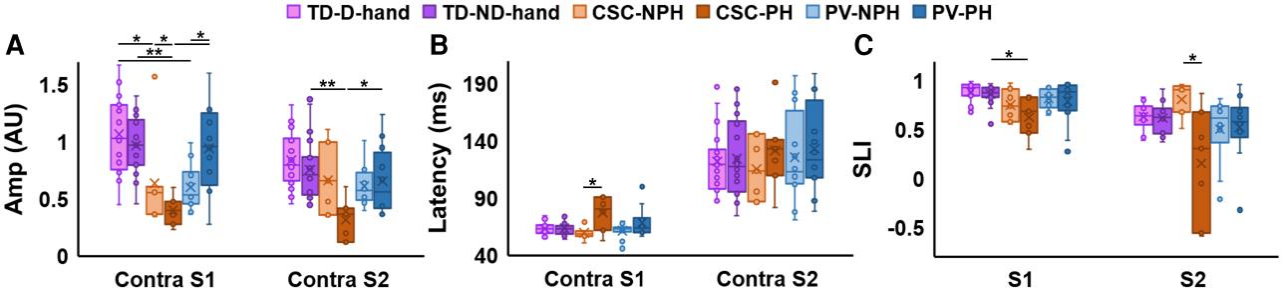
**Somatosensory evoked potential amplitudes, latencies, and lateralization.** Box-and-whisker plots of contralateral primary and secondary somatosensory (S1 and S2, respectively) peak amplitude (arbitrary units; **A**), peak latency (ms; **B**) and somatosensory lateralization index (SLI; **C**) in response to haptic stimulation of middle finger of dominant (TD-D-hand) and non-dominant (TD-ND-hand) hands of typically developing (TD; *N* = 24) controls and the paretic (PH) and non-paretic (NPH) hands of cortical-subcortical (CSC; *N* = 7) and periventricular (PV; *N* = 12) groups. Boxes indicate median and interquartile range, whiskers range, × denotes mean, and o represents individual data points for each participant. *P < 0.05, **P < 0.01, ***P < 0.001 derived from Wilcoxon signed rank tests for within subject, and Kruskal–Wallis and Mann–Whitney *U* tests for between subject comparisons (FDR corrected for multiple comparisons).

Stimulation of the PH in the CSC group resulted in suppressed contralateral SER amplitudes in S1 (*P* *<* 0.01, r = 0.84) and S2 (*P* *<* 0.01, r = 0.88) compared with ND-hand of TD controls and PH of PV group (Fig. 4A). Additionally, the NPHs of both CSC and PV groups showed decreased contralesional S1 activation relative to the TD group (*P* *<* 0.05, r > 0.75). The CSC group displayed prolonged contralateral S1 latency in response to stimulation of the PH compared to their NPH (*P* *<* 0.05, r = 0.89; Fig. 4B). Stimulation of the PH in the CSC group resulted in reduced S1 lateralization (*P* *<* 0.05, r = 0.81) compared to TD participants (Fig. 4C). Additionally, S2 activation was higher in the NPH compared to the PH within the CSC group (*P* *<* 0.05, r = 0.89).

### Tractography

#### Ascending sensory fibres

**Figure 5:**
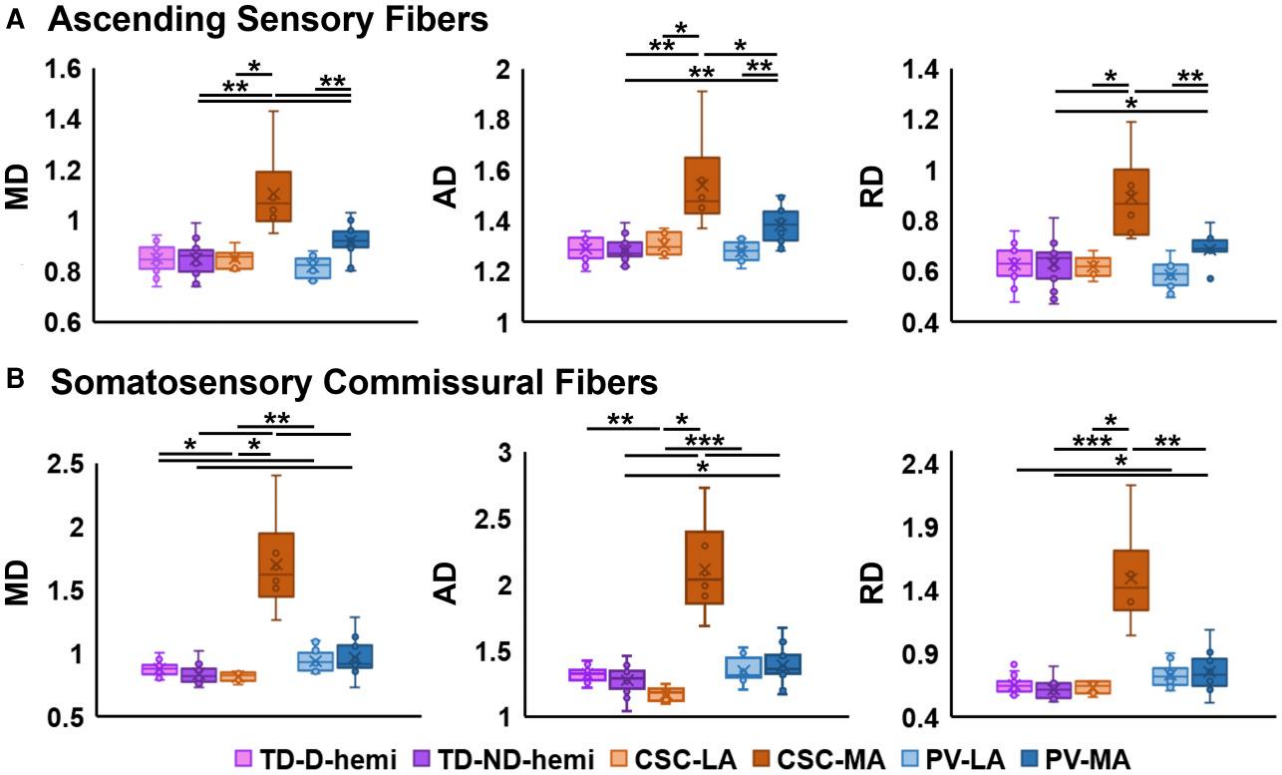
**Diffusivity metrics in somatosensory white matter tracts.** Box-and-whisker plots of mean diffusivity (MD), axial diffusivity (AD) and radial diffusivity (RD) in ascending sensory fibres (ASF; **A**) and somatosensory commissural fibres (SCF; **B**) for the dominant (TD-D-hemi) and non-dominant (TD-ND-hemi) hemispheres of typically developing (TD; *N* = 22) controls and the less affected (LA) and more affected (MA) hemispheres of cortical-subcortical (CSC; *N* = 6) and periventricular (PV; *N* = 12) groups. Boxes indicate median and interquartile range, whiskers range, × denotes mean, and o represents individual data points for each participant. *P < 0.05, **P < 0.01, ***P < 0.001 derived from Wilcoxon signed rank tests for within subject, and Kruskal–Wallis and Mann–Whitney *U* tests for between-subject comparisons (FDR corrected for multiple comparisons).

MD was elevated in the MA hemisphere of both CSC and PV groups when compared to their corresponding LA hemispheres (CSC: *P* *<* 0.05, r = 0.90; PV: *P* *<* 0.01, r = 0.86) and ND-hemi of the TD group (CSC: *P* *<* 0.01, r = 0.47; PV: *P* *<* 0.01, r = 0.25; Fig. 5A). AD and RD showed similar differences among three groups (see Fig. 5A). This observation reflects a completely unilateral microstructural damage to ASF in both lesion types with greater severity in CSC than PV cases (MD: *P* *<* 0.01, r = 2.43; RD: *P* *<* 0.01, r = 2.43; AD: *P* *<* 0.05, r = 2.25).

#### Somatosensory commissural fibre

MD was elevated in the MA hemisphere of CSC children compared to their LA (*P* *<* 0.05, r = 0.90) and ND-hemi of TD group (*P* *<* 0.01, r = 0.48; Fig. 5B). The PV group showed increased MD in the both hemispheres relative to the TD group (*P* *<* 0.05, r = 0.12) and in the LA hemisphere compared to the LA hemisphere of the CSC group (*P* *<* 0.01, r = 0.99). AD was also higher in the MA hemisphere of CSC participants when compared to its LA (*P* *<* 0.05, r = 0.90) and ND-hemi of TD children (*P* *<* 0.001, r = 0.48). Interestingly, the AD in the CSC group’s LA hemisphere was lower than D-hemi of the TD group (*P* *<* 0.01, r = 0.91) and the LA of the PV group (*P* *<* 0.001, r = 0.95). RD was higher in the CSC group’s MA hemisphere compared to its LA side (*P* *<* 0.05, r = 0.90) and ND-hemi of TD group (*P* *<* 0.001, r = 0.48). The PV group also showed elevated RD bilaterally compared to the TD group (*P* *<* 0.05, r = 0.08). These findings confirm SCF microstructural damage in the MA hemisphere of the CSC group and both hemispheres of the PV group, with greater severity in the CSC group (MD: *P* *<* 0.01, r = 2.50; RD: *P* *<* 0.01, r = 2.50; AD: *P* *<* 0.001, r = 2.52). Further detailed results of all statistical comparisons are reported in Supplementary Table 2.

### Intermodality associations

#### Functional measures—behavioural outcomes

Reduced S1 and S2 amplitudes were strongly associated with poorer tactile function. S1 amplitude correlated with touch sensitivity (*Rho* = −0.60, *P* *<* 0.05; Table 2) and two-point discrimination (*Rho* = −0.77, *P* *<* 0.05). Prolonged latency in S1 correlated with higher touch thresholds (*Rho* = 0.63, *P* *<* 0.05). Lateralization indices in both S1 and S2 were negatively correlated with discrimination thresholds (S1 LI: *Rho* = −0.73, *P* *<* 0.05; S2 LI: *Rho* = −0.55, *P* *<* 0.05).

**Table 2:** Intermodality correlations among structural, functional and behavioural metrics

Metric #1	Metric #2	All subjects and sides		MA/PH of HCP groups	
		Spearman’s Rho	*P*-value (FDR)	Spearman’s Rho	*P*-value (FDR)
Lesion size	Contra S2 amp	-	-	−0.55	0.048
Contra S1 amp	Touch sensitivity	−0.31	0.023	−0.60	0.042
Contra S1 amp	2-point discrimination	−0.52	3.59E-05	−0.77	0.027
Contra S2 amp	Touch sensitivity	−0.24	0.079	−0.56	0.036
Contra S2 amp	2-point discrimination	−0.32	0.015	−0.43	0.135
Contra S1 latency	Touch sensitivity	0.07	0.595	0.63	0.036
S1 LI	2-point discrimination	−0.43	0.002	−0.73	0.028
S2 LI	2-point discrimination	−0.22	0.096	−0.55	0.048
ASF MD	Touch sensitivity	0.35	0.014	0.72	0.029
ASF AD	Touch sensitivity	0.34	0.014	0.75	0.027
ASF RD	Touch sensitivity	0.38	0.008	0.63	0.029
ASF MD	Contra S1 amp	−0.06	0.93	−0.55	0.048
ASF MD	Contra S1 latency	0.15	0.25	0.56	0.036
ASF AD	Contra S1 latency	0.19	0.160	0.69	0.029
ASF MD	Contra S2 amp	−0.21	0.121	−0.60	0.036
ASF AD	Contra S2 amp	−0.40	0.008	−0.55	0.048
ASF MD	S2 LI	−0.20	0.148	−0.71	0.029
ASF AD	S2 LI	−0.34	0.016	−0.64	0.029
ASF RD	S2 LI	−0.17	0.207	−0.68	0.029
SCF MD	Touch sensitivity	0.22	0.117	0.72	0.029
SCF AD	Touch sensitivity	0.23	0.100	0.69	0.029
SCF RD	Touch sensitivity	0.21	0.133	0.74	0.028
SCF MD	2-point discrimination	0.38	0.008	0.52	0.056
SCF AD	2-point discrimination	0.35	0.014	0.67	0.029
SCF MD	Contra S1 amp	−0.20	0.148	−0.77	0.025
SCF AD	Contra S1 amp	−0.20	0.148	−0.78	0.025
SCF RD	Contra S1 amp	−0.22	0.117	−0.76	0.025
SCF MD	Contra S2 amp	−0.27	0.056	−0.66	0.029
SCF AD	Contra S2 amp	−0.22	0.121	−0.58	0.042
SCF RD	Contra S2 amp	−0.29	0.035	−0.67	0.029
SCF AD	S1 LI	−0.13	0.57	−0.72	0.029
SCF MD	S2 LI	−0.34	0.014	−0.54	0.055
SCF AD	S2 LI	−0.35	0.014	−0.41	0.129
SCF RD	S2 LI	−0.35	0.014	−0.62	0.032

AD, axial diffusivity; amp, amplitude; ASF, ascending sensory fibre; FDR, false discovery rate; HCP, hemiplegic cerebral palsy; LI, lateralization index; MA, more affected; MD, mean diffusivity; PH, paretic hand; RD, radial diffusivity; S1, primary somatosensory cortex; S2, secondary somatosensory cortex; SCF, somatosensory commissural fibre.

*P*-values obtained from Spearman’s correlation and corrected for multiple comparisons (FDR). *P*-values < 0.05 are represented in bold.

#### Microstructural measures—behavioural outcomes

For ASF, touch sensitivity was positively correlated with MD (*Rho* = 0.72, *P* *<* 0.05), AD (*Rho* = 0.75, *P* *<* 0.05), and RD (*Rho* = 0.63, *P* *<* 0.05). For SCF, MD, AD and RD were strongly correlated with both behavioural measures—for example, RD with touch sensitivity (*Rho* = 0.74, *P* *<* 0.05), and AD with discrimination (*Rho* = 0.67, *P* *<* 0.05). For full report of the significant correlations see Table 2.

#### Microstructural—functional measures

For the ASF, contralateral S1 amplitude was negatively linked to MD (*Rho* = −0.55, *P* *<* 0.05), and contralateral S1 latency was moderately and positively correlated with MD (*Rho* = 0.56, *P* *<* 0.05) and AD (*Rho* = 0.69, *P* *<* 0.05). Contralateral S2 amplitude also negatively correlated with MD (*Rho* = −0.60, *P* *<* 0.05) and AD (*Rho* = −0.55, *P* *<* 0.05). S2 latency, however, did not show a significant correlation with ASF metrics. Regarding interhemispheric balance, higher diffusivity within the ASF was associated with reduced functional lateralization in S2. Specifically, MD (*Rho* = −0.71, *P* *<* 0.05), AD (*Rho* = −0.64, *P* *<* 0.05) and RD (*Rho* = −0.68, *P* *<* 0.05) each showed strong negative correlations with the S2 lateralization index. No significant correlation was observed between ASF diffusion metrics and S1 lateralization index.

For SCF, contralateral S1 amplitude was strongly correlated with the MD (*Rho* = −0.77, *P* *<* 0.05), AD (*Rho* = −0.78, *P* *<* 0.05) and RD (*Rho* = −0.76, *P* *<* 0.05). Contralateral S2 amplitude showed weaker associations with SCF diffusivity metrics: it was negatively associated with MD (*Rho* = −0.66, *P* *<* 0.05), AD (*Rho* = −0.58, *P* *<* 0.05) and RD (*Rho* = −0.67, *P* *<* 0.05). No significant correlations were observed between S1/S2 latency and SCF diffusivity metrics. Lastly, S1 lateralization was inversely related to AD (*Rho* = −0.72, *P* *<* 0.05). Similarly, S2 lateralization was negatively correlated with RD (*Rho* = −0.62, *P* *<* 0.05).

## Discussion

By capitalizing on advanced multimodal neuroimaging, we show that children with HCP exhibit distinct structural and functional neuroplasticity patterns based on their lesion type. Early PV lesions primarily affect the white matter, sparing cortical structures, and thereby allowing for greater potential for adaptive neuroplasticity during development, which results in relatively preserved sensorimotor function. In contrast, late CSC lesions involve direct damage to both cortical and subcortical structures, thereby disrupting essential somatosensory regions and substantially limiting the capacity for adaptive neuroplasticity; severe deficits in sensorimotor outcomes manifest these disruptions. Our findings provide new insights into the neuroplasticity mechanism in HCP and highlight potential targets for optimizing therapeutic interventions.

### CSC and PV lesions cause distinguishable structural damage

CSC lesions disproportionately affected the grey matter and resulted in more extensive volume loss. Most CSC lesions, caused by middle cerebral artery stroke, affected pre- and post-central gyri, damaging both S1 and S2. One of the key consequences of this structural damage to S1 and S2 was the suppressed SER in the MA hemisphere. This may be due to neuronal necrosis caused by hypoxic-ischemic injury.^47^ In addition, larger lesions, especially in the CSC group, were linked to diminished ability in touch sensitivity and discrimination.

The effects of the insult to specific brain structures were reflected in outcomes as well. Damage to grey matter (i.e. neuronal necrosis) in CSC lesions may eliminate both intra-regional information processing (in S1 and S2) and inter-regional information transfer (across ASF and SCF). These structural damages may disable the processing of afferent information to S1 (also represented as suppressed SER amplitude) and disable the transmitting capabilities of the sensorimotor network (represented as delayed SER).

Somatosensory behavioural results can also be utilized to better contrast between effects of cortical versus subcortical grey matter damage. In line with previous studies,^11,18,23,24^ PV lesions with minimal (or no) cortical damage showed minimal touch sensitivity deficits; this is possibly due to close to normal low-order sensory processing in S1. Whereas CSC lesions led to severely diminished, or in some cases, complete lack of touch sensitivity. Both lesion types showed deficits in discrimination, more severe in the CSC group. This can be associated with prevalent damage to the thalamus and basal ganglia, especially in the CSC group.^48-50^ Poor (or complete) lack of discriminability in the CSC group can also be associated with the lesion damaging the S2, a crucial hub in the high-order somatosensory network.^51^

### CSC and PV lesions cause distinguishable damage to ASF and SCF

Further illustrating the neuroplasticity potential of children with HCP, tractography showed that ASF can bypass the lesion, projecting to corresponding cortical areas in the MA hemisphere of all children with PV and most children with CSC lesions. Furthermore, microstructural analysis of ASF revealed unilateral damage in the MA hemisphere of both groups, with MD and AD being higher, and more pronounced damage in the CSC group. Increased MD may be caused by gliosis and microscopic cystic degeneration,^52^ while increased AD may indicate lower axonal density and axonal loss.^53^ As a measure of water diffusion perpendicular to fibres, RD was also elevated, particularly in the CSC group, implying possible demyelination, cerebral oedema, or myelin pathology.^54^ These diffusivity values in the MA hemisphere of the CSC group were associated with poor touch sensitivity. This relationship highlights the role of ASF, as the first transmitter of afferent inputs, and its axonal integrity in lower-order somatosensory processing. In contrast, ASF diffusivity metrics were not linked to two-point discrimination. This distinction suggests that higher-order somatosensory functions involve more complex and distributed processing pathways, making them less directly tied to afferent tract integrity alone.

Similar intergroup variations in structural integrity were observed in the SCF, with disruptions evident in the MA hemisphere of the CSC group and bilaterally in the PV group. Radiological evaluations also indicated more prevalent damage to the posterior CC body in the PV group. This pattern suggests that the bilateral rise in MD, AD and RD in the PV group likely indicates arrested SCF maturation, possibly caused by damaged myelination following early prenatal injury.^55^ Higher diffusivity metrics were also linked to both low- and high-order sensory impairments, which may implicate the involvement of interhemispheric pathways in somatosensory processing.^49,56^

Previous studies have shown a relationship between CC structural integrity and outcome.^57^ These studies also reported that HCP children with lower levels of CC damage possess a higher potential for upper-extremity function improvement following rehabilitation.^58^ This highlights the importance of preserved interhemispheric connectivity for higher-order somatosensory processing.^59^ In our cohort, disruptions in interhemispheric integration appeared to reflect distinct lesion-related patterns of white matter damage. CSC lesions cause more severe disruption to ASF and SCF fibres in the MA hemisphere. On the other hand, PV lesions primarily affect the ASF unilaterally and SCF bilaterally. The developmental timing of these lesions may explain this pattern. PV lesions precede the ASF development therefore allowing these fibres to bypass the lesion and thus lessen the damage degree. In addition, since CC fibres develop before ASF,^60^ PV lesions are more likely to occur during SCF development which leads to damages to CC and bilateral SCF. Contrarily, CSC lesions occur during (or after) ASF development and after SCF development, increasing the fibres’ susceptibility to direct damage. These findings highlight how lesion type and timing shape somatosensory outcomes in HCP, with late CSC lesions causing direct cortical damage and greater disruption to both afferent processing and intra- and interhemispheric connectivity. In contrast, early PV lesions often spare cortical areas and permit adaptive reorganization, particularly for lower-order functions.

Our tractography findings build directly on and extend the tract-level results reported by Gupta *et al*.^11^ While Gupta *et al*. evaluated the medial lemniscus and demonstrated reduced integrity in children with cortical lesions, our study identifies an additional and previously unrecognized substrate of sensory dysfunction, i.e. the somatosensory commissural fibres. Disruption of these commissural pathways was strongly associated with reduced S1/S2 lateralization, revealing an interhemispheric abnormality that is not assessed in previous works.

### CSC lesions disrupt afferent signal transmission and processing

S1 was localized in the post-central gyrus of the MA hemisphere in most HCP participants. In two participants of the CSC group, the peak amplitude of the first SER component was localized in the subcortical grey matter, near the lesion. These two participants also showed a lack of touch sensitivity and two-point discrimination. Evoked activity in contralateral S1 was suppressed in both hands of the CSC group and the NPH of the PV group. These observations contrast with the expected diminished S1 amplitude in response to only the PH stimulation. Instead, they possibly reveal a disruption in the mediation of information flow and processing in S1.^61^ This pattern may reflect that damage to deep structures leads to suppressed S1 activity in both hemispheres of children with CSC lesion.^62^ In contrast, S1 amplitude in the MA hemisphere of the PV group was comparable to TD. Although unexpected, this finding may reflect functional neuroplasticity and the compensatory engagement of spared cortex to offset the disrupted input and damage to the ASF.^61^

A robust association was found between S1 amplitude responses and ASF/SCF diffusion metrics. In ASF, MD was correlated negatively with contralateral S1 amplitude, possibly implying that axonal loss (or demyelination) compromises the afferent fibres’ capacity to transmit action potentials and activate S1, thereby reducing cortical responsiveness. In SCF, MD, AD and RD were correlated negatively with S1 amplitude. These associations reveal a link between S1 activity— as the first cortical hub for lower- and higher-order somatosensory processing— and commissural fibres’ structural integrity.^63^ Reduced S1 amplitude was associated with elevated touch sensitivity and two-point discrimination threshold; this indicates that diminished neuronal activation in S1 is tied to impaired sensory processing.^64^ This was particularly evident for the PH of children with CSC lesions, where evoked responses were often severely attenuated and behavioural performance was poorest.

The prolonged S1 latency in the MA hemisphere of the CSC group likely reflects impaired conduction through ASF due to disrupted microstructural integrity. These delayed S1 responses were strongly associated with elevated MD and AD in these fibres. This reinforces the interpretation that compromised axonal density and myelination slow action potential transmission and degrade the temporal dynamics of cortical activation.^65^ Notably, the latency of contralateral S1 responses also correlated with higher sensory thresholds. These delays likely reduce the precision of sensory processing timing, contributing to diminished perceptual acuity.^64^ However, when examining higher-order sensory processing, the complexity and heterogeneity of parallel information pathways introduce considerable variability.^61^ As a result, the latency of second peaks in cortical evoked responses showed high variance across all subjects.

While our results are consistent with the previous literature on somatosensory cortical activity,^11^ our study reveals several new physiological signatures not previously reported. These results highlight a distinct cortical pathophysiology that was not characterized in previous studies. For instance, Gupta *et al*.^11^ established that cortical lesions relate to greater sensory impairment manifested as a lack of SEP in response to vibrotactile stimulation, our findings reveal the underlying physiological mechanisms: suppressed S1/S2 responsiveness and slowed S1 temporal processing. Together, these metrics illustrate a pattern of disrupted thalamocortical integration and reduced interhemispheric coordination, providing mechanistic insight into why CSC lesions produce more severe sensory deficits than PV lesions.

### CSC lesions disrupt the interhemispheric balance

We have previously reported ipsilateral S1 and S2 activities in response to haptic stimulation in TD children.^66^ These activities were less prominent than those in the contralateral hemisphere, establishing an ‘interhemispheric balance’ in typical somatosensory processing. Here, we quantified this balance with SLIs of S1 and S2. TD children exhibited high SLI in S1 bilaterally, reflecting strong lateralization and minimal involvement of the ipsilateral hemisphere in low-order somatosensory processing. In contrast, the CSC group showed lower SLI in S1 in response to PH stimulation. This suggests increased involvement of ipsilateral S1 and a shift in early sensory processing. In S2, TD children demonstrated lower SLI consistent with typical bilateral involvement in high-order processing.^50^ The abnormally low SLI in S2 during PH stimulation in the CSC group suggests disrupted interhemispheric equilibrium and heightened contribution of contralesional S2, possibly due to reorganization of sensory processing pathways.^67^

Diffusivity metrics were inversely associated with SLIs in both S1 and S2. Higher MD, AD and RD in ASF were linked to S2 SLI. This is due to impaired afferent signal transmission, possibly disrupting serial and parallel somatosensory processing in S2 bilaterally.^66^ In addition, alterations in SCF diffusivity parameters were related to decreased S1 and S2 lateralization. This suggests that white matter injury, particularly in the commissural fibres, may drive the atypical bilateral processing observed in our functional analyses, further linking microstructural damage to maladaptive reorganization. Another possible cause for the effect of SCF damage on imbalanced SLI is the disrupted inhibitory role of the ipsilateral S1 in somatosensory processing.^68,69^ Previous work has suggested that somatosensory deficits in HCP, particularly in the proprioceptive domain, may in part reflect an altered excitation–inhibition balance within the somatosensory cortices. Nurmi *et al*.^70^ reported that stronger proprioceptive-evoked responses in somatosensory regions were associated with worse sensorimotor function, which they interpreted as reflecting reduced inhibitory control and abnormal cortical gain. Findings from animal models are congruent with this interpretation, demonstrating that neonatal hypoxia leads to enlarged receptive fields and increased neuronal responsiveness in the primary somatosensory cortex.^71^ In addition, post-mortem studies of neonates with perinatal brain injury have reported loss or disruption of GABAergic inhibitory interneurons, suggesting that early injury-related alterations to inhibitory circuitry may have long-lasting effects on somatosensory cortical organization and function.^72,73^

Reduced SLI in S1 and S2 correlated strongly with behavioural deficits. Lower SLIs—indicating greater reliance on ipsilateral or bilateral processing—were associated with impaired discriminability.^67^ This pattern was particularly evident in the CSC group, suggesting that a shift away from typical contralateral dominance (interhemispheric balance) may reflect maladaptive reorganization that fails to support functional recovery. The strengthened involvement of contralesional S2 after CSC lesions might reflect the emergence of potential alternative pathways in the absence of ipsilesional S2. Yet, a prevalent interhemispheric dissociation between S1 and S2 in response to afferent somatosensory inputs limits this potential. These findings demonstrate that CSC lesions disrupt typical interhemispheric balance by reducing S1 and S2 lateralization and increasing bilateral activation, linking microstructural white matter damage to maladaptive sensory reorganization and poorer discrimination. Ultimately, the observed bilateral somatosensory representation in the reorganized brain of children with CSC lesions may impact treatment decisions, especially regarding direct invasive or non-invasive brain stimulation targets.

### Limitations and future directions

Due to the heterogeneity and lower prevalence of other aetiologies in HCP (different brain malformations), we were not able to classify these participants in a coherent group. Furthermore, the small sample size, especially in the CSC group, may limit the generalizability of our findings. A comprehensive study with a larger sample size may address these limitations. Due to the cross-sectional design, we also cannot directly assess developmental trajectories of neuroplasticity. Longitudinal studies are needed to explore how these functional and structural patterns evolve with age and intervention. Finally, further exploration of secondary somatosensory areas (e.g. S2 connectivity) and cortical-subcortical interactions may provide additional insights into compensatory mechanisms.

## Conclusion

This study elucidates distinct neuroplasticity profiles in children with CSC and PV lesions. CSC lesions are characterized by more severe cortical and subcortical damage, with limited compensatory reorganization. In contrast, PV lesions involve less direct cortical injury, allowing for more effective structural and functional adaptation. Notably, pronounced contralesional activation of S1 and S2 in response to haptic stimulation of the PH of the CSC group suggests some retained neuroplasticity potential. These findings underscore the importance of early detection and intervention—such as somatosensory training—for children with CSC lesions to improve sensory and motor outcomes. Altogether, our findings highlight the importance of lesion-specific treatment plans and the benefit of combining structural and functional neuroimaging to guide tailored rehabilitation programmes. These revelations deepen our knowledge of adaptive neuroplasticity in paediatric HCP and open the path for enhancing sensorimotor results utilizing precision medicine strategies.

## Supplementary Material

fcag185_Supplementary_Data

## Data Availability

The data are available from the corresponding author upon request. The code is available on GitHub (https://github.com/shsadra/Brain-Communications-2025.git).

## References

[1] Heest AEV, House J, Putnam M. Sensibility deficiencies in the hands of children with spastic hemiplegia. J Hand Surg Am. 1993;18(2):278–281. 8463594 10.1016/0363-5023(93)90361-6

[2] Cooper J, Majnemer A, Rosenblatt B, Birnbaum R. The determination of sensory deficits in children with hemiplegic cerebral palsy. J Child Neurol. 1995;10(4):300–309. 7594266 10.1177/088307389501000412

[3] Sanger TD, Kukke SN. Abnormalities of tactile sensory function in children with dystonic and diplegic cerebral palsy. J Child Neurol. 2007;22(3):289–293. 17621498 10.1177/0883073807300530

[4] Wingert JR, Burton H, Sinclair RJ, Brunstrom JE, Damiano DL. Joint-position sense and kinesthesia in cerebral palsy. Arch Phys Med Rehabil. 2009;90(3):447–453. 19254610 10.1016/j.apmr.2008.08.217 PMC2651562

[5] Arner M, Eliasson AC, Nicklasson S, Sommerstein K, Hägglund G. Hand function in cerebral palsy. Report of 367 children in a population-based longitudinal health care program. J Hand Surg Am. 2008;33(8):1337–1347. 18929198 10.1016/j.jhsa.2008.02.032

[6] Wiklund LM, Uvebrant P. Hemiplegic cerebral palsy: Correlation between CT morphology and clinical findings. Dev Med Child Neurol. 1991;33(6):512–523. 1864477 10.1111/j.1469-8749.1991.tb14916.x

[7] Gordon AM, Bleyenheuft Y, Steenbergen B. Pathophysiology of impaired hand function in children with unilateral cerebral palsy. Dev Med Child Neurol. 2013;55(Suppl 4):32–37. 24237277 10.1111/dmcn.12304

[8] Rosenbaum P, Paneth N, Leviton A, et al A report: The definition and classification of cerebral palsy April 2006. Dev Med Child Neurol Suppl. 2007;109(suppl 109):8–14. 17370477

[9] Staudt M. (Re-)organization of the developing human brain following periventricular white matter lesions. Neurosci Biobehav Rev. 2007;31(8):1150–1156. 17624432 10.1016/j.neubiorev.2007.05.005

[10] Staudt M. Reorganization after pre- and perinatal brain lesions. J Anat. 2010;217(4):469–474. 20649910 10.1111/j.1469-7580.2010.01262.x PMC2992421

[11] Gupta D, Barachant A, Gordon AM, et al Effect of sensory and motor connectivity on hand function in pediatric hemiplegia. Ann Neurol. 2017;82(5):766–780. 29034483 10.1002/ana.25080 PMC5708868

[12] Papadelis C, Ahtam B, Nazarova M, et al Cortical somatosensory reorganization in children with spastic cerebral palsy: A multimodal neuroimaging study. Front Hum Neurosci. 2014;8:725. 25309398 10.3389/fnhum.2014.00725 PMC4162364

[13] Papadelis C, Butler EE, Rubenstein M, et al Reorganization of the somatosensory cortex in hemiplegic cerebral palsy associated with impaired sensory tracts. Neuroimage Clin. 2018;17:198–212. 29159037 10.1016/j.nicl.2017.10.021 PMC5683344

[14] Papadelis C, Ahtam B, Feldman HA, et al Altered white matter connectivity associated with intergyral brain disorganization in hemiplegic cerebral palsy. Neuroscience. 2019;399:146–160. 30593919 10.1016/j.neuroscience.2018.12.028 PMC10716912

[15] Kolb B, Mychasiuk R, Muhammad A, Gibb R. Chapter 2—Brain plasticity in the developing brain. In: Merzenich MM, Nahum M, Vleet TMV, eds. Changing brains. Vol 207. Progress in Brain Research. Elsevier; 2013:35–64. 10.1016/B978-0-444-63327-9.00005-9 24309250

[16] Kolb B, Muhammad A, Gibb R. Searching for factors underlying cerebral plasticity in the normal and injured brain. J Commun Disord. 2011;44(5):503–514. 21621219 10.1016/j.jcomdis.2011.04.007

[17] Jaspers E, Byblow WD, Feys H, Wenderoth N. The corticospinal tract: A biomarker to categorize upper limb functional potential in unilateral cerebral palsy. Front Pediatr. 2016;3:112. 26779464 10.3389/fped.2015.00112 PMC4701904

[18] Knijnenburg ACS, Steinbusch CVM, Janssen-Potten YJM, Defesche A, Vermeulen RJ. Neuro-imaging characteristics of sensory impairment in cerebral palsy; a systematic review. Front Rehabil Sci. 2023;4:1084746. 37009398 10.3389/fresc.2023.1084746 PMC10065191

[19] Ballabh P, de Vries LS. White matter injury in infants with intraventricular haemorrhage: Mechanisms and therapies. Nat Rev Neurol. 2021;17(4):199–214. 33504979 10.1038/s41582-020-00447-8 PMC8880688

[20] Jiang H, Li X, Jin C, et al Early diagnosis of spastic cerebral palsy in infants with periventricular white matter injury using diffusion tensor imaging. AJNR Am J Neuroradiol. 2019;40(1):162–168. 30545838 10.3174/ajnr.A5914 PMC7048607

[21] Giotta Lucifero A, Baldoncini M, Bruno N, et al Microsurgical neurovascular anatomy of the brain: The anterior circulation (Part I). Acta Biomed. 2021;92(S4):e2021412. 34437363 10.23750/abm.v92iS4.12116 PMC9179062

[22] Reid SM, Dagia CD, Ditchfield MR, Reddihough DS. Grey matter injury patterns in cerebral palsy: Associations between structural involvement on MRI and clinical outcomes. Developmental Medicine & Child Neurology. 2015;57(12):1159–1167. 25970144 10.1111/dmcn.12800

[23] Feys H, Eyssen M, Jaspers E, et al Relation between neuroradiological findings and upper limb function in hemiplegic cerebral palsy. Eur J Paediatr Neurol. 2010;14(2):169–177. 19272822 10.1016/j.ejpn.2009.01.004

[24] Fehlings D, Krishnan P, Ragguett RM, et al Neurodevelopmental profiles of children with unilateral cerebral palsy associated with middle cerebral artery and periventricular venous infarctions. Developmental Medicine & Child Neurology. 2021;63(6):729–735. 33521966 10.1111/dmcn.14818 PMC8247945

[25] Reid LB, Rose SE, Boyd RN. Rehabilitation and neuroplasticity in children with unilateral cerebral palsy. Nat Rev Neurol. 2015;11(7):390–400. 26077839 10.1038/nrneurol.2015.97

[26] Jaatela J, Nurmi T, Vallinoja J, Mäenpää H, Sairanen V, Piitulainen H. Altered corpus callosum structure in adolescents with cerebral palsy: Connection to gait and balance. Brain Struct Funct. 2023;228(8):1901–1915. 37615759 10.1007/s00429-023-02692-1 PMC10516810

[27] Nevalainen P, Pihko E, Mäenpää H, Valanne L, Nummenmaa L, Lauronen L. Bilateral alterations in somatosensory cortical processing in hemiplegic cerebral palsy. Dev Med Child Neurol. 2012;54(4):361–367. 22211315 10.1111/j.1469-8749.2011.04165.x

[28] Song Y, Renoul E, Acord S, et al Aberrant somatosensory phase synchronization in children with hemiplegic cerebral palsy. Neurosci Lett. 2021;762:136169. 34390772 10.1016/j.neulet.2021.136169 PMC8882340

[29] Brun C, Traverse É, Granger É, Mercier C. Somatosensory deficits and neural correlates in cerebral palsy: A scoping review. Dev Med Child Neurol. 2021;63(12):1382–1393. 34145582 10.1111/dmcn.14963 PMC9290873

[30] Corona L, Rijal S, Tanritanir O, et al Electromagnetic source imaging in presurgical evaluation of children with drug-resistant epilepsy. J Vis Exp. 2024(211):e66494. 10.3791/66494 PMC11512582 39373494

[31] Mahmutoglu MA, Rupp A, Baumgärtner U. Simultaneous EEG/MEG yields complementary information of nociceptive evoked responses. Clinical Neurophysiology. 2022;143:21–35. 36087398 10.1016/j.clinph.2022.08.005

[32] Bast T, Wright T, Boor R, et al Combined EEG and MEG analysis of early somatosensory evoked activity in children and adolescents with focal epilepsies. Clinical neurophysiology. 2007;118(8):1721–1735. 17572142 10.1016/j.clinph.2007.03.037

[33] Suda M, Kawakami M, Okuyama K, et al Validity and reliability of the Semmes-Weinstein monofilament test and the thumb localizing test in patients with stroke. Front Neurol. 2021;11:625917. 33584520 10.3389/fneur.2020.625917 PMC7873561

[34] Dellon AL, Mackinnon SE, Crosby PM. Reliability of two-point discrimination measurements. J Hand Surg Am. 1987;12(5):693–696. 3655225 10.1016/s0363-5023(87)80049-7

[35] Randall M, Johnson L, Reddihough D. The Melbourne assessment 2. Royal Children’s Hospital; 1999.

[36] Ahlfors SP, Han J, Belliveau JW, Hämäläinen MS. Sensitivity of MEG and EEG to source orientation. Brain Topogr. 2010;23(3):227–232. 20640882 10.1007/s10548-010-0154-x PMC2914866

[37] Espenhahn S, Rossiter HE, van Wijk BCM, et al Sensorimotor cortex beta oscillations reflect motor skill learning ability after stroke. Brain Commun. 2020;2(2):fcaa161. 33215085 10.1093/braincomms/fcaa161 PMC7660041

[38] Tadel F, Baillet S, Mosher JC, Pantazis D, Leahy RM. Brainstorm: A user-friendly application for MEG/EEG analysis. Comput Intell Neurosci. 2011;2011(1):879716. 21584256 10.1155/2011/879716 PMC3090754

[39] Tesche CD, Uusitalo MA, Ilmoniemi RJ, Huotilainen M, Kajola M, Salonen O. Signal-space projections of MEG data characterize both distributed and well-localized neuronal sources. Electroencephalogr Clin Neurophysiol. 1995;95(3):189–200. 7555909 10.1016/0013-4694(95)00064-6

[40] Delorme A, Makeig S. EEGLAB: An open source toolbox for analysis of single-trial EEG dynamics including independent component analysis. J Neurosci Methods. 2004;134(1):9–21. 15102499 10.1016/j.jneumeth.2003.10.009

[41] Dale AM, Liu AK, Fischl BR, et al Dynamic statistical parametric mapping: Combining fMRI and MEG for high-resolution imaging of cortical activity. Neuron. 2000;26(1):55–67. 10798392 10.1016/s0896-6273(00)81138-1

[42] Hämäläinen MS, Ilmoniemi RJ. Interpreting magnetic fields of the brain: Minimum norm estimates. Med Biol Eng Comput. 1994;32:35–42. 8182960 10.1007/BF02512476

[43] Gramfort A, Papadopoulo T, Olivi E, Clerc M. OpenMEEG: Opensource software for quasistatic bioelectromagnetics. Biomed Eng Online. 2010;9:45. 20819204 10.1186/1475-925X-9-45 PMC2949879

[44] Seghier ML. Laterality index in functional MRI: Methodological issues. Magn Reson Imaging. 2008;26(5):594–601. 18158224 10.1016/j.mri.2007.10.010 PMC2726301

[45] Yeh FC, Wedeen VJ, Tseng WYI. Generalized ${q} $-sampling imaging. IEEE Trans Med Imaging. 2010;29(9):1626–1635. 20304721 10.1109/TMI.2010.2045126

[46] Yeh FC, Panesar S, Barrios J, et al Automatic removal of false connections in diffusion MRI tractography using topology-informed pruning (TIP). Neurotherapeutics. 2019;16(1):52–58. 30218214 10.1007/s13311-018-0663-y PMC6361061

[47] Volpe JJ. Neurology of the Newborn. 1987:236–279. 7022034

[48] Fabri M, Burton H. Ipsilateral cortical connections of primary somatic sensory cortex in rats. J Comp Neurol. 1991;311(3):405–424. 1720147 10.1002/cne.903110310

[49] Coghill RC, Gilron I, Iadarola MJ. Hemispheric lateralization of somatosensory processing. J Neurophysiol. 2001;85(6):2602–2612. 11387404 10.1152/jn.2001.85.6.2602

[50] Pala A, Stanley GB. Ipsilateral stimulus encoding in primary and secondary somatosensory cortex of awake mice. Journal of Neuroscience. 2022;42(13):2701–2715. 35135855 10.1523/JNEUROSCI.1417-21.2022 PMC8973421

[51] Zilles K, Palomero-Gallagher N. 4.14—The Architecture of somatosensory Cortex. In: Fritzsch B, ed. The senses: A comprehensive reference (second edition). 2nd Ed. Elsevier; 2020:225–260.

[52] Neil JJ, Shiran SI, McKinstry RC, et al Normal brain in human newborns: Apparent diffusion coefficient and diffusion anisotropy measured by using diffusion tensor MR imaging. Radiology. 1998;209(1):57–66. 9769812 10.1148/radiology.209.1.9769812

[53] Sun SW, Liang HF, Cross AH, Song SK. Evolving Wallerian degeneration after transient retinal ischemia in mice characterized by diffusion tensor imaging. Neuroimage. 2008;40(1):1–10. 18187343 10.1016/j.neuroimage.2007.11.049 PMC2276530

[54] Song SK, Yoshino J, Le TQ, et al Demyelination increases radial diffusivity in corpus callosum of mouse brain. Neuroimage. 2005;26(1):132–140. 15862213 10.1016/j.neuroimage.2005.01.028

[55] Volpe JJ. Brain injury in premature infants: A complex amalgam of destructive and developmental disturbances. Lancet Neurol. 2009;8(1):110–124. 19081519 10.1016/S1474-4422(08)70294-1 PMC2707149

[56] Ragert P, Nierhaus T, Cohen LG, Villringer A. Interhemispheric interactions between the human primary somatosensory cortices. PLoS One. 2011;6(2):e16150. 21347308 10.1371/journal.pone.0016150 PMC3037378

[57] Gooijers J, Swinnen SP. Interactions between brain structure and behavior: The corpus callosum and bimanual coordination. Neurosci Biobehav Rev. 2014;43:1–19. 24661987 10.1016/j.neubiorev.2014.03.008

[58] Robert MT, Gutterman J, Ferre CL, et al Corpus callosum integrity relates to improvement of upper-extremity function following intensive rehabilitation in children with unilateral spastic cerebral palsy. Neurorehabil Neural Repair. 2021;35(6):534–544. 33955304 10.1177/15459683211011220 PMC8135240

[59] Tzourio-Mazoyer N. Intra- and inter-hemispheric connectivity supporting hemispheric specialization. In: Micro-, meso-and macro-connectomics of the brain. Springer; 2016:129–146. 28590670

[60] Wang R, Wilkinson M, Kane T, Takahashi E. Convergence of cortical, thalamocortical, and callosal pathways during human fetal development revealed by diffusion MRI tractography. Front Neurosci. 2017;11:576. 29163000 10.3389/fnins.2017.00576 PMC5671991

[61] Xie K, Royer J, Rodriguez-Cruces R, et al Temporal lobe epilepsy perturbs the brain-wide excitation-inhibition balance: Associations with microcircuit organization, clinical parameters, and cognitive dysfunction. Advanced Science. 2025;12:2406835. 39806576 10.1002/advs.202406835 PMC11884548

[62] Szczupak D, Iack PM, Liu C, et al Direct interhemispheric cortical communication via thalamic commissures: A new white-matter pathway in the rodent brain. Cerebral Cortex. 2021;31(10):4642–4651. 33999140 10.1093/cercor/bhab112 PMC8408456

[63] Chung S, Bacon T, Rath JF, et al Callosal interhemispheric communication in mild traumatic brain injury: A mediation analysis on WM microstructure effects. AJNR Am J Neuroradiol. 2024;45(6):788–794. 38637026 10.3174/ajnr.A8213 PMC11288603

[64] Allison T, McCarthy G, Wood CC, Jones SJ. Potentials evoked in human and monkey cerebral cortex by stimulation of the median nerve: A review of scalp and intracranial recordings. Brain. 1991;114(6):2465–2503. 1782527 10.1093/brain/114.6.2465

[65] Hamann J, Ettrich B, Hoffman KT, Then Bergh F, Lobsien D. Somatosensory evoked potentials and their relation to microstructural damage in patients with multiple sclerosis—A whole brain DTI study. Front Neurol. 2022;13:890841. 36105776 10.3389/fneur.2022.890841 PMC9465089

[66] Song Y, Shahdadian S, Armstrong E, et al Spatiotemporal dynamics of cortical somatosensory network in typically developing children. Cerebral Cortex. 2024;34(6):bhae230. 38836408 10.1093/cercor/bhae230 PMC11151116

[67] Lemée JM, Chinier E, Ali P, Labriffe M, Minassian AT, Dinomais M. (Re)organisation of the somatosensory system after early brain lesion: A lateralization index fMRI study. Ann Phys Rehabil Med. 2020;63(5):416–421. 30825646 10.1016/j.rehab.2019.02.001

[68] Hirano M, Kimoto Y, Shiotani S, Furuya S. Enhanced somatosensory inhibition sharpens hand representation and sensorimotor skills in pianists. J Neurosci. 2025;45(8). 10.1523/JNEUROSCI.1486-24.2024 PMC11841757 39746821

[69] Sutherland MT. The hand and the ipsilateral primary somatosensory cortex. Journal of Neuroscience. 2006;26(32):8217–8218. 16906643 10.1523/JNEUROSCI.2698-06.2006 PMC6673806

[70] Nurmi T, Jaatela J, Vallinoja J, Mäenpää H, Piitulainen H. Stronger proprioceptive BOLD-responses in the somatosensory cortices reflect worse sensorimotor function in adolescents with and without cerebral palsy. Neuroimage Clin. 2021;32:102795. 34474316 10.1016/j.nicl.2021.102795 PMC8411230

[71] Coq JO, Strata F, Russier M, et al Impact of neonatal asphyxia and hind limb immobilization on musculoskeletal tissues and S1 map organization: Implications for cerebral palsy. Exp Neurol. 2008;210(1):95–108. 18061167 10.1016/j.expneurol.2007.10.006

[72] Robinson S, Li Q, DeChant A, Cohen ML. Neonatal loss of γ–aminobutyric acid pathway expression after human perinatal brain injury. J Neurosurg Pediatr. 2006;104(6):396–408. 10.3171/ped.2006.104.6.396 PMC1762128 16776375

[73] Stolp HB, Fleiss B, Arai Y, et al Interneuron development is disrupted in preterm brains with diffuse white matter injury: Observations in mouse and human. Front Physiol. 2019;10:955. 31417418 10.3389/fphys.2019.00955 PMC6683859

