# Supplementary material for: Functional and structural neuroplasticity of somatosensory system in hemiplegic cerebral palsy
Source: Brain Commun. 2026 May 21;8(3):fcag185. doi: 10.1093/braincomms/fcag185 (PMC13403283; doi:10.1093/braincomms/fcag185)
Supplement: fcag185_Supplementary_Data [file fcag185_Supplementary_Data.pdf]

Supplementary Table 1 Behavioral, functional, and structural results for the 'other' lesion type

		#10		#11		#16	
**Metric**		**NPH**	**PH**	**NPH**	**PH**	**NPH**	**PH**
**Behavioral**	**Touch sensitivity (g)**	0.07	0.07	0.07	0.16	0.07	0.16
	**Two-point discrimination (mm)**	2	2	2	3	3	10
	**Range of motion (%)**	100	92.6	100	90.6	100	83.3
	**Accuracy (%)**	100	100	100	96.3	100	86.4
	**Dexterity (%)**	100	75	100	93.8	100	38.5
	**Fluency (%)**	100	85.7	100	76.2	100	66.7
**Functional**	**Contra S1 amp (AU)**	0.72	0.17	0.98	0.39	0.51	0.85
	**Contra S1 latency (ms)**	65	75	58	57	89	86
	**S1 LI**	0.7	0.98	0.68	-0.11	0.70	0.23
	**Contra S2 amp (AU)**	1.02	0.39	0.38	0.97	0.68	0.37
	**Contra S2 latency (ms)**	174	142	111	136	140	141
	**S2 LI**	0.49	0.09	-0.2	0.89	0.57	0.11
		**LA**	**MA**	**LA**	**MA**	**LA**	**MA**
**Structural**	**ASF MD**	0.79	0.82	0.76	0.77	0.86	1.03
	**ASF AD**	1.29	1.35	1.18	1.30	1.33	1.49
	**ASF RD**	0.53	0.55	0.55	0.51	0.62	0.79
	**SCF MD**	0.81	0.75	0.89	0.75	1.28	1.20
	**SCF AD**	1.34	1.29	1.35	1.23	1.68	1.67
	**SCF RD**	0.55	0.48	0.66	0.51	1.09	0.96

AD = axial diffusivity; amp = amplitude; ASF = ascending sensory fiber; LA = less affected; LI = lateralization index; MA = more affected; MD = mean diffusivity; NPH = non-paretic hand; PH = paretic hand; RD = radial diffusivity; S1 = primary somatosensory cortex; S2 = secondary somatosensory cortex; SCF = somatosensory commissural fiber.

Supplementary Table 2 Results of statistical comparisons performed

Metric	Comparison	Statistical Test	Raw P	Adjusted P	Effect Size r
**D1 Sensitivity**	CSC_NPH vs CSC_PH	Wilcoxon signed-rank	0.05906	0.1575	0.894
	PV_NPH vs PV_PH	Wilcoxon signed-rank	0.233	0.4568	0.804
	CSC_NPH vs TD_D-hand	Mann-Whitney	0.7364	0.7364	0.169
	PV_NPH vs TD_D-hand	Mann-Whitney	0.7364	0.7364	0.484
	PV_NPH vs CSC_NPH	Mann-Whitney	0.7364	0.7364	1.548
	CSC_PH vs TD_ND-hand	Mann-Whitney	0.003093	**0.02474**	0.302
	PV_PH vs TD_ND-hand	Mann-Whitney	0.2855	0.4568	0.261
	PV_PH vs CSC_PH	Mann-Whitney	0.01615	0.0446	2.075
**D3 Sensitivity**	CSC_NPH vs CSC_PH	Wilcoxon signed-rank	0.05906	0.1181	0.894
	PV_NPH vs PV_PH	Wilcoxon signed-rank	0.007914	**0.03166**	0.885
	CSC_NPH vs TD_D-hand	Mann-Whitney	0.4155	0.4156	0.063
	PV_NPH vs TD_D-hand	Mann-Whitney	0.4156	0.4156	0.549
	PV_NPH vs CSC_NPH	Mann-Whitney	0.4155	0.4156	1.718
	CSC_PH vs TD_ND-hand	Mann-Whitney	0.001972	**0.01578**	0.34
	PV_PH vs TD_ND-hand	Mann-Whitney	0.1059	0.1694	0.167
	PV_PH vs CSC_PH	Mann-Whitney	0.01321	**0.03523**	2.007
**D5 Sensitivity**	CSC_NPH vs CSC_PH	Wilcoxon signed-rank	0.03603	0.07206	0.894

Supplementary Table 2 (continued) Results of statistical comparisons performed

Metric	Comparison	Statistical Test	Raw P	Adjusted P	Effect Size r
	PV_NPH vs PV_PH	Wilcoxon signed-rank	0.03103	0.07206	0.885
	CSC_NPH vs TD_D-hand	Mann-Whitney	0.3741	0.3741	0.081
	PV_NPH vs TD_D-hand	Mann-Whitney	0.3741	0.3741	0.541
	PV_NPH vs CSC_NPH	Mann-Whitney	0.3741	0.3741	1.622
	CSC_PH vs TD_ND-hand	Mann-Whitney	0.000927	**0.007412**	0.382
	PV_PH vs TD_ND-hand	Mann-Whitney	0.252	0.3741	0.219
	PV_PH vs CSC_PH	Mann-Whitney	0.005711	**0.02285**	2.071
**All Sensitivity**	CSC_NPH vs CSC_PH	Wilcoxon signed-rank	0.000719	**0.001439**	0.877
	PV_NPH vs PV_PH	Wilcoxon signed-rank	0.000298	**0.000794**	0.846
	CSC_NPH vs TD_D-hand	Mann-Whitney	0.2495	0.2495	0.102
	PV_NPH vs TD_D-hand	Mann-Whitney	0.2495	0.2495	0.489
	PV_NPH vs CSC_NPH	Mann-Whitney	0.2495	0.2495	1.572
	CSC_PH vs TD_ND-hand	Mann-Whitney	1.32E-08	**1.06E-07**	0.363
	PV_PH vs TD_ND-hand	Mann-Whitney	0.02853	**0.04565**	0.193
	PV_PH vs CSC_PH	Mann-Whitney	6.93E-06	2.77E-05	2.014
**D1 2-point**	CSC_NPH vs CSC_PH	Wilcoxon signed-rank	0.03603	0.07206	0.891
	PV_NPH vs PV_PH	Wilcoxon signed-rank	0.08897	0.1424	0.885
	CSC_NPH vs TD_D-hand	Mann-Whitney	0.73	0.8342	0.165
	PV_NPH vs TD_D-hand	Mann-Whitney	0.73	0.8342	0.243
	PV_NPH vs CSC_NPH	Mann-Whitney	0.8574	0.8574	1.203
	CSC_PH vs TD_ND-hand	Mann-Whitney	7.72E-05	**0.000618**	0.392
	PV_PH vs TD_ND-hand	Mann-Whitney	0.000932	**0.003728**	0.067
	PV_PH vs CSC_PH	Mann-Whitney	0.0309	0.07206	1.794
**D3 2-point**	CSC_NPH vs CSC_PH	Wilcoxon signed-rank	0.02154	**0.04308**	0.891
	PV_NPH vs PV_PH	Wilcoxon signed-rank	0.1058	0.1231	0.818
	CSC_NPH vs TD_D-hand	Mann-Whitney	0.1077	0.1231	0.004
	PV_NPH vs TD_D-hand	Mann-Whitney	0.03916	0.06265	0.03
	PV_NPH vs CSC_NPH	Mann-Whitney	0.8147	0.8147	1.193
	CSC_PH vs TD_ND-hand	Mann-Whitney	2.33E-05	**0.000186**	0.439
	PV_PH vs TD_ND-hand	Mann-Whitney	0.000769	**0.003076**	0.126
	PV_PH vs CSC_PH	Mann-Whitney	0.006709	**0.01789**	1.866
**D5 2-point**	CSC_NPH vs CSC_PH	Wilcoxon signed-rank	0.02201	**0.03522**	0.891
	PV_NPH vs PV_PH	Wilcoxon signed-rank	0.1696	0.2261	0.831
	CSC_NPH vs TD_D-hand	Mann-Whitney	0.4178	0.4178	0.15
	PV_NPH vs TD_D-hand	Mann-Whitney	0.01402	**0.02805**	0.036
	PV_NPH vs CSC_NPH	Mann-Whitney	0.2707	0.3094	0.957
	CSC_PH vs TD_ND-hand	Mann-Whitney	0.000104	**0.000834**	0.412
	PV_PH vs TD_ND-hand	Mann-Whitney	0.00493	**0.01972**	0.051
	PV_PH vs CSC_PH	Mann-Whitney	0.0134	**0.02805**	1.809
**All 2-point**	CSC_NPH vs CSC_PH	Wilcoxon signed-rank	9.02E-05	**0.00018**	0.875
	PV_NPH vs PV_PH	Wilcoxon signed-rank	0.004349	**0.005798**	0.838

Supplementary Table 2 (continued) Results of statistical comparisons performed

Metric	Comparison	Statistical Test	Raw P	Adjusted P	Effect Size r
	CSC_NPH vs TD_D-hand	Mann-Whitney	0.08001	0.09144	0.087
	PV_NPH vs TD_D-hand	Mann-Whitney	0.000732	**0.001172**	0.06
	PV_NPH vs CSC_NPH	Mann-Whitney	0.2558	0.2558	1.063
	CSC_PH vs TD_ND-hand	Mann-Whitney	6.15E-13	**4.92E-12**	0.433
	PV_PH vs TD_ND-hand	Mann-Whitney	3.4E-08	**1.36E-07**	0.098
	PV_PH vs CSC_PH	Mann-Whitney	1.65E-05	**4.41E-05**	1.777
**ROM**	CSC_NPH vs CSC_PH	Wilcoxon signed-rank	0.02225	0.05934	0.894
	PV_NPH vs PV_PH	Wilcoxon signed-rank	0.05917	0.1037	0.43
	CSC_NPH vs TD_D-hand	Mann-Whitney	0.6434	0.6434	0.208
	PV_NPH vs TD_D-hand	Mann-Whitney	0.4542	0.5191	0.534
	PV_NPH vs CSC_NPH	Mann-Whitney	0.4542	0.5191	1.648
	CSC_PH vs TD_ND-hand	Mann-Whitney	6.06E-06	**4.84E-05**	0.95
	PV_PH vs TD_ND-hand	Mann-Whitney	0.00998	**0.03992**	0.783
	PV_PH vs CSC_PH	Mann-Whitney	0.06482	0.1037	1.086
**Accuracy**	CSC_NPH vs CSC_PH	Wilcoxon signed-rank	0.03603	0.09608	0.447
	PV_NPH vs PV_PH	Wilcoxon signed-rank	0.1148	0.1838	0.464
	CSC_NPH vs TD_D-hand	Mann-Whitney	0.7429	0.7429	0.344
	PV_NPH vs TD_D-hand	Mann-Whitney	0.7429	0.7429	0.501
	PV_NPH vs CSC_NPH	Mann-Whitney	0.7429	0.7429	1.444
	CSC_PH vs TD_ND-hand	Mann-Whitney	0.001348	**0.01078**	0.781
	PV_PH vs TD_ND-hand	Mann-Whitney	0.09936	0.1838	0.663
	PV_PH vs CSC_PH	Mann-Whitney	0.03172	0.09608	0.989
**Dexterity**	CSC_NPH vs CSC_PH	Wilcoxon signed-rank	0.01563	**0.03125**	0.894
	PV_NPH vs PV_PH	Wilcoxon signed-rank	0.02977	**0.04764**	0.113
	CSC_NPH vs TD_D-hand	Mann-Whitney	0.4542	0.5191	0.059
	PV_NPH vs TD_D-hand	Mann-Whitney	0.5796	0.5796	0.366
	PV_NPH vs CSC_NPH	Mann-Whitney	0.4542	0.5191	1.648
	CSC_PH vs TD_ND-hand	Mann-Whitney	2.6E-06	**2.08E-05**	0.95
	PV_PH vs TD_ND-hand	Mann-Whitney	0.000797	**0.003186**	0.878
	PV_PH vs CSC_PH	Mann-Whitney	0.001583	**0.00422**	0.785
**Fluency**	CSC_NPH vs CSC_PH	Wilcoxon signed-rank	0.05906	0.07382	0.064
	PV_NPH vs PV_PH	Wilcoxon signed-rank	0.03603	0.06005	0.408
	CSC_PH vs TD_ND-hand	Mann-Whitney	3.42E-05	**0.000171**	0.747
	PV_PH vs TD_ND-hand	Mann-Whitney	0.000329	**0.000822**	0.839
	PV_PH vs CSC_PH	Mann-Whitney	0.2187	0.2187	1.231
**S1 amp**	CSC_NPH vs CSC_PH	Wilcoxon signed-rank	0.2969	0.3958	0.447
	PV_NPH vs PV_PH	Wilcoxon signed-rank	0.009277	**0.01677**	0.725
	CSC_NPH vs TD_D-hand	Mann-Whitney	0.01048	**0.01677**	0.748
	PV_NPH vs TD_D-hand	Mann-Whitney	0.000735	**0.00294**	1.081
	PV_NPH vs CSC_NPH	Mann-Whitney	0.5821	0.6653	1.377
	CSC_PH vs TD_ND-hand	Mann-Whitney	0.000474	**0.00294**	0.944

Supplementary Table 2 (continued) Results of statistical comparisons performed

Metric	Comparison	Statistical Test	Raw P	Adjusted P	Effect Size r
	PV_PH vs TD_ND-hand	Mann-Whitney	0.7942	0.7942	0.505
	PV_PH vs CSC_PH	Mann-Whitney	0.00604	**0.01611**	0.843
**S1 latency**	CSC_NPH vs CSC_PH	Wilcoxon signed-rank	0.01563	0.125	0.894
	PV_NPH vs PV_PH	Wilcoxon signed-rank	0.5743	0.6564	0.396
	CSC_NPH vs TD_D-hand	Mann-Whitney	0.2069	0.4138	0.513
	PV_NPH vs TD_D-hand	Mann-Whitney	0.84	0.84	0.473
	PV_NPH vs CSC_NPH	Mann-Whitney	0.2069	0.4138	1.163
	CSC_PH vs TD_ND-hand	Mann-Whitney	0.09397	0.3759	0.153
	PV_PH vs TD_ND-hand	Mann-Whitney	0.4798	0.6397	0.316
	PV_PH vs CSC_PH	Mann-Whitney	0.3038	0.486	1.813
**S1 LI**	CSC_NPH vs CSC_PH	Wilcoxon signed-rank	0.3096	0.4689	0.415
	PV_NPH vs PV_PH	Wilcoxon signed-rank	0.8445	0.8533	0.068
	CSC_NPH vs TD_D-hand	Mann-Whitney	0.07002	0.1867	0.618
	PV_NPH vs TD_D-hand	Mann-Whitney	0.07002	0.1867	0.805
	PV_NPH vs CSC_NPH	Mann-Whitney	0.3517	0.4689	1.289
	CSC_PH vs TD_ND-hand	Mann-Whitney	0.004929	**0.03943**	0.806
	PV_PH vs TD_ND-hand	Mann-Whitney	0.8533	0.8533	0.47
	PV_PH vs CSC_PH	Mann-Whitney	0.0943	0.1886	1.076
**S2 amp**	CSC_NPH vs CSC_PH	Wilcoxon signed-rank	0.03125	0.0625	0.831
	PV_NPH vs PV_PH	Wilcoxon signed-rank	0.5186	0.5926	0.204
	CSC_NPH vs TD_D-hand	Mann-Whitney	0.143	0.1907	0.56
	PV_NPH vs TD_D-hand	Mann-Whitney	0.01452	0.05665	0.937
	PV_NPH vs CSC_NPH	Mann-Whitney	0.9326	0.9326	1.541
	CSC_PH vs TD_ND-hand	Mann-Whitney	0.001074	**0.008594**	0.882
	PV_PH vs TD_ND-hand	Mann-Whitney	0.1224	0.1907	0.696
	PV_PH vs CSC_PH	Mann-Whitney	0.02124	0.05665	0.94
**S2 latency**	CSC_NPH vs CSC_PH	Wilcoxon signed-rank	0.5534	1	0.256
	PV_NPH vs PV_PH	Wilcoxon signed-rank	0.3278	1	0.396
	CSC_NPH vs TD_D-hand	Mann-Whitney	1	1	0.318
	PV_NPH vs TD_D-hand	Mann-Whitney	1	1	0.433
	PV_NPH vs CSC_NPH	Mann-Whitney	1	1	1.435
	CSC_PH vs TD_ND-hand	Mann-Whitney	0.7996	1	0.136
	PV_PH vs TD_ND-hand	Mann-Whitney	0.7996	1	0.341
	PV_PH vs CSC_PH	Mann-Whitney	0.7996	1	1.58
**S2 LI**	CSC_NPH vs CSC_PH	Wilcoxon signed-rank	0.01563	0.125	0.894
	PV_NPH vs PV_PH	Wilcoxon signed-rank	0.9697	0.9697	0.023
	CSC_NPH vs TD_D-hand	Mann-Whitney	0.07039	0.1877	0.127

Supplementary Table 2 (continued) Results of statistical comparisons performed

Metric	Comparison	Statistical Test	Raw P	Adjusted P	Effect Size r
	PV_NPH vs TD_D-hand	Mann-Whitney	0.3058	0.4078	0.61
	PV_NPH vs CSC_NPH	Mann-Whitney	0.07039	0.1877	1.978
	CSC_PH vs TD_ND-hand	Mann-Whitney	0.1576	0.3151	0.59
	PV_PH vs TD_ND-hand	Mann-Whitney	0.4399	0.5028	0.568
	PV_PH vs CSC_PH	Mann-Whitney	0.285	0.4078	1.202
**Lesion size**	PV_MA vs CSC_MA	Mann-Whitney	0.04601	**0.04601**	1.953
**ASF-MD**	CSC_LA vs CSC_MA	Wilcoxon signed-rank	0.03125	**0.048**	0.899
	PV_LA vs PV_MA	Wilcoxon signed-rank	0.000977	**0.003906**	0.861
	CSC_LA vs TD_D-hemi	Mann-Whitney	0.9329	0.9329	0.243
	PV_LA vs TD_D-hemi	Mann-Whitney	0.306	0.3497	0.742
	PV_LA vs CSC_LA	Mann-Whitney	0.306	0.3497	2.031
	CSC_MA vs TD_ND-hemi	Mann-Whitney	0.000902	**0.003906**	0.466
	PV_MA vs TD_ND-hemi	Mann-Whitney	0.003278	**0.006555**	0.025
	PV_MA vs CSC_MA	Mann-Whitney	0.003278	**0.006555**	2.428
**ASF-AD**	CSC_LA vs CSC_MA	Wilcoxon signed-rank	0.03552	0.05684	0.899
	PV_LA vs PV_MA	Wilcoxon signed-rank	0.003252	**0.008671**	0.861
	CSC_LA vs TD_D-hemi	Mann-Whitney	0.4693	0.4693	0.053
	PV_LA vs TD_D-hemi	Mann-Whitney	0.4693	0.4693	0.609
	PV_LA vs CSC_LA	Mann-Whitney	0.4693	0.4693	1.943
	CSC_MA vs TD_ND-hemi	Mann-Whitney	0.000431	**0.001726**	0.466
	PV_MA vs TD_ND-hemi	Mann-Whitney	0.000329	**0.001726**	0.182
	PV_MA vs CSC_MA	Mann-Whitney	0.02758	0.05515	2.252
**ASF-RD**	CSC_LA vs CSC_MA	Wilcoxon signed-rank	0.03125	**0.047**	0.899
	PV_LA vs PV_MA	Wilcoxon signed-rank	0.002516	**0.01007**	0.883
	CSC_LA vs TD_D-hemi	Mann-Whitney	0.6333	0.6333	0.317
	PV_LA vs TD_D-hemi	Mann-Whitney	0.2129	0.2839	0.794
	PV_LA vs CSC_LA	Mann-Whitney	0.3315	0.3788	2.02
	CSC_MA vs TD_ND-hemi	Mann-Whitney	0.001357	**0.01007**	0.444
	PV_MA vs TD_ND-hemi	Mann-Whitney	0.02511	**0.045**	0.096
	PV_MA vs CSC_MA	Mann-Whitney	0.0046	**0.01227**	2.428
**SCF-MD**	CSC_LA vs CSC_MA	Wilcoxon signed-rank	0.03125	**0.03839**	0.899
	PV_LA vs PV_MA	Wilcoxon signed-rank	0.7334	0.7334	0.113

Supplementary Table 2 (continued) Results of statistical comparisons performed

Metric	Comparison	Statistical Test	Raw P	Adjusted P	Effect Size r
	CSC_LA vs TD_D-hemi	Mann-Whitney	0.02563	**0.03839**	0.667
	PV_LA vs TD_D-hemi	Mann-Whitney	0.03359	**0.03839**	0.117
	PV_LA vs CSC_LA	Mann-Whitney	0.002262	**0.004525**	0.993
	CSC_MA vs TD_ND-hemi	Mann-Whitney	1.59E-05	**0.000127**	0.476
	PV_MA vs TD_ND-hemi	Mann-Whitney	0.001802	**0.004525**	0.037
	PV_MA vs CSC_MA	Mann-Whitney	0.000323	**0.001293**	2.495
**SCF-AD**	CSC_LA vs CSC_MA	Wilcoxon signed-rank	0.03125	**0.04167**	0.899
	PV_LA vs PV_MA	Wilcoxon signed-rank	0.5693	0.6507	0.181
	CSC_LA vs TD_D-hemi	Mann-Whitney	3.19E-05	**0.000127**	0.91
	PV_LA vs TD_D-hemi	Mann-Whitney	0.6829	0.6829	0.408
	PV_LA vs CSC_LA	Mann-Whitney	0.000323	**0.000646**	0.949
	CSC_MA vs TD_ND-hemi	Mann-Whitney	1.59E-05	**0.000127**	0.476
	PV_MA vs TD_ND-hemi	Mann-Whitney	0.0227	**0.03632**	0.093
	PV_MA vs CSC_MA	Mann-Whitney	0.000162	**0.000431**	2.517
**SCF-RD**	CSC_LA vs CSC_MA	Wilcoxon signed-rank	0.03125	**0.04167**	0.899
	PV_LA vs PV_MA	Wilcoxon signed-rank	0.9097	0.9097	0.045
	CSC_LA vs TD_D-hemi	Mann-Whitney	0.8487	0.9097	0.265
	PV_LA vs TD_D-hemi	Mann-Whitney	0.02731	**0.04167**	0.049
	PV_LA vs CSC_LA	Mann-Whitney	0.02731	**0.04167**	1.17
	CSC_MA vs TD_ND-hemi	Mann-Whitney	1.59E-05	**0.000127**	0.476
	PV_MA vs TD_ND-hemi	Mann-Whitney	0.004071	**0.01086**	0
	PV_MA vs CSC_MA	Mann-Whitney	0.000323	**0.001293**	2.495

AD = axial diffusivity; amp = amplitude; ASF = ascending sensory fiber; D-hand = dominant hand; D-hemi = dominant hemisphere; LA = less affected; CSD = cortical-subcortical LI = lateralization index; MA = more affected; MD = mean diffusivity; ND-hand = non-dominant hand; ND-hemi = non-dominant hemisphere; NPH = non-paretic hand; PH = paretic hand; PV = periventricular; RD = radial diffusivity; S1 = primary somatosensory cortex; S2 = secondary somatosensory cortex; SCF = somatosensory commissural fiber; TD = typically developing. P-values are corrected for multiple comparison using FDR correction.
